# Self-Sealing Complex Oxide Resonators

**DOI:** 10.1021/acs.nanolett.1c03498

**Published:** 2022-02-04

**Authors:** Martin Lee, Martin P. Robin, Ruben H. Guis, Ulderico Filippozzi, Dong Hoon Shin, Thierry C. van Thiel, Stijn P. Paardekooper, Johannes R. Renshof, Herre S. J. van der Zant, Andrea D. Caviglia, Gerard J. Verbiest, Peter G. Steeneken

**Affiliations:** † Kavli Institute of Nanoscience, Delft University of Technology, Lorentzweg 1, 2628 CJ Delft, The Netherlands; ‡ Department of Precision and Microsystems Engineering, Delft University of Technology, Mekelweg 2, 2628 CD Delft, The Netherlands

**Keywords:** Nanomechanics, Membranes, Complex oxides, Perovskites, Pressure sensors, NEMS

## Abstract

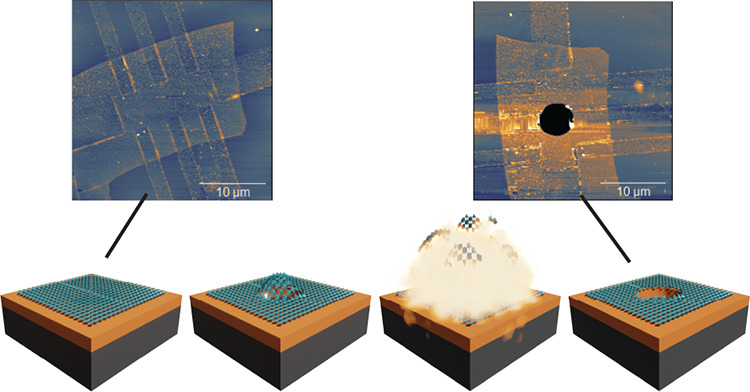

Although 2D materials hold great potential for next-generation pressure sensors, recent studies revealed that gases permeate along the membrane-surface interface, necessitating additional sealing procedures. In this work, we demonstrate the use of free-standing complex oxides as self-sealing membranes that allow the reference cavity beneath to be sealed by a simple anneal. To test the hermeticity, we study the gas permeation time constants in nanomechanical resonators made from SrRuO_3_ and SrTiO_3_ membranes suspended over SiO_2_/Si cavities which show an improvement up to 4 orders of magnitude in the permeation time constant after annealing the devices. Similar devices fabricated on Si_3_N_4_/Si do not show such improvements, suggesting that the adhesion increase over SiO_2_ is mediated by oxygen bonds that are formed at the SiO_2_/complex oxide interface during the self-sealing anneal. Picosecond ultrasonics measurements confirm the improvement in the adhesion by 70% after annealing.

## Introduction

van der Waals (vdW) materials attracted significant attention in the microelectromechanical systems (MEMS) community due to their low dimensionality, flexibility, and strength.^1^ In particular, graphene is considered as the material for the next generation pressure sensors^2−7^ thanks to its intrinsic impermeability to gases.^8−10^ Pressure sensors operate by measuring the deflection of a membrane due to the pressure difference between a reference cavity and the environment. For a reliable pressure sensor, hermeticity of the cavity underneath the membrane is essential. However, gas permeation along the interface between the vdW membrane and the substrate causes pressure variations in the reference cavity, which renders the pressure readings from graphene-based pressure sensors unreliable.^11,12^

Recently reported sealing protocols have enabled improvements in the hermeticity of vdW material membranes of up to a factor 10 000,^11,12^ but scaling them to high volume production is difficult, since depositing and patterning of sealing layers on top of ultrathin vdW material membranes is often detrimental to device performance in particular if high temperatures are needed. Moreover, the pressure at which the sealing layer is deposited is often fixed by the process, such that the reference pressure in the cavity cannot be freely controlled.^13−16^

As an alternative to graphene, we introduce in this letter, free-standing single crystal complex oxide perovskites as a membrane for MEMS applications. Unlike vdW materials, which are fundamentally at a disadvantage due to the inevitability of the weak vdW interaction with the substrate, complex oxides are able to form chemical bonds with the substrate at high temperatures.^17^ Therefore, complex oxides promise a stronger adhesion to the substrate than 2D materials. For example, using direct wafer bonding (DWB), single crystal complex oxides such as LiNbO_3_ have been shown to be able to bond directly onto other substrates like Si^18−20^ to create submicron thick MEMS resonators.^21−25^ Moreover, due to their epitaxial crystalline growth, extremely uniform surfaces are grown using pulsed laser deposition (PLD), enhancing the interface contact^26^ and reducing the formation probability of gas leakage pathways. Recent developments in releasing epitaxially grown single crystal complex oxides allow them to be thinned down to the unit cell limit, similar to the vdW materials.^27−29^ Complex oxides in their ultrathin free-standing form are mechanically robust^30^ while withstanding strains up to 8%,^31,32^ are flexible enough to allow large curvatures^33^ and have already been demonstrated as viable nanomechanical resonators.^34,35^ Furthermore, wafer-scale production of thin-film single crystalline complex oxides are being developed^36^ which makes them even more appealing for large-scale CMOS compatible fabrication.

Here, we use free-standing SrRuO_3_ (SRO) and SrTiO_3_ (STO) suspended over SiO_2_/Si cavities to make pressure sensors and demonstrate a simple, CMOS compatible sealing technique similar to DWB, which does not require additional fabrication steps. The sealing consists of annealing the devices above 300 °C in ambient conditions for 15 min. We measure the time dependence of the resonance frequency to extract the gas permeation time constant. By comparing the permeation time constant of the pressure sensor devices before and after performing the self-sealing annealing process, we show that the permeation time constant increases from 14 s to >10 000 s, indicative of a large increase in hermeticity. Comparable devices fabricated on Si_3_N_4_/Si cavities do not show such enhancement of the hermeticity which suggests that the improved hermeticity is mediated by the properties of the SiO_2_ that promote adhesion to the complex oxides, thus eliminating gas leakage rates. Furthermore, we probe the STO–SiO_2_ interface in both annealed and nonannealed samples using a picosecond ultrasonics technique. The measurements show a clear reduction in the ultrasonic reflection coefficient at the interface between STO-SiO_2_ after the annealing procedure, indicative of an increased adhesion. Our work investigates the use of ultrathin complex oxide membranes for pressure sensors and demonstrates self-sealing of the interface after transfer, thus providing an alternative to graphene and MEMS sensor technologies.

## Results and Discussion

**Figure 1:**
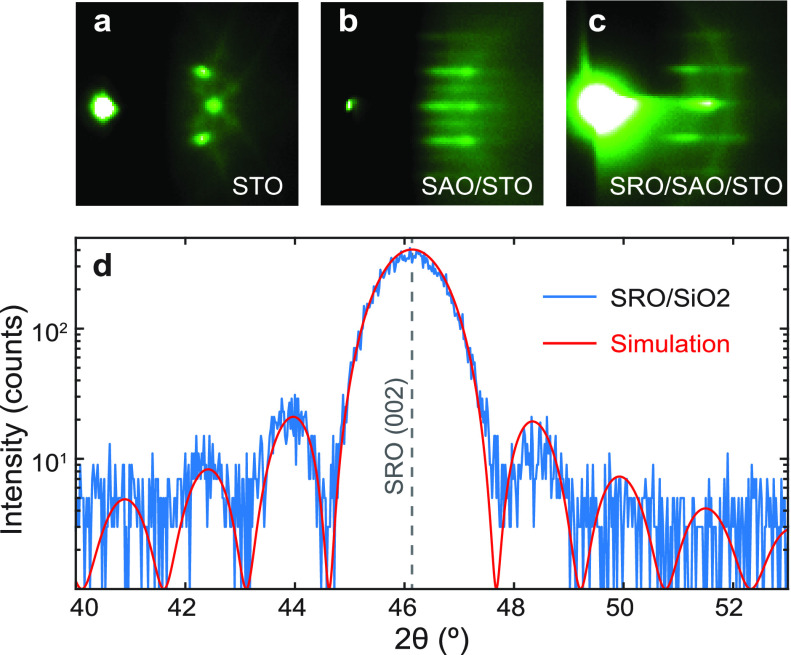
Reflection high-energy electron diffraction (RHEED) images of (a) SrTiO_3_ (STO) substrate, (b) Sr_3_Al_2_O_6_ (SAO) grown on STO substrate, and (c) SrRuO_3_ grown on SAO/STO stack. (d) X-ray diffraction (blue) of exfoliated SRO stamped on SiO_2_/Si and the simulation (red). The *c*-axis lattice parameter extracted from the simulation is 3.931 Å, and the thickness is 16 unit cells.

Crystalline free-standing complex oxides are synthesized using PLD by growing a (water-soluble) buffer layer of Sr_3_Al_2_O_6_ (SAO) on SrTiO_3_ (001) substrates, followed by an overlayer of choice (STO or SRO). The growth is monitored by in situ reflection high energy electron diffraction (RHEED), confirming 2D growth (Figure 1a–c). After growth, the samples are attached to polydimethylsiloxane (PDMS) films for support during the etching process of the buffer layer which is performed by submerging the PDMS covered sample in deionized water for 24 h. After the SAO is etched away, the film of choice is transferred onto a dummy SiO_2_/Si substrate using a deterministic transfer method^37^ for characterization. See Supporting Information (Sections S.I–S.III).

We perform X-ray diffraction (XRD) measurements on the films after transferring parts of them to a dummy SiO_2_/Si to verify the film thicknesses and the crystal coherence. As shown in Figure 1d, the crystallographic (002) peak of SRO is identified with finite-size oscillations on both sides of the main peak, showing long-range crystal coherence of the film after exfoliation and the transfer process. A model fit plotted in red on top of the XRD data is used to extract the *c*-axis lattice parameter as well as the number of pseudocubic unit cells. In the case of SRO (Figure 1d), the model yields a thickness of 16 unit cells (u.c.) with a *c*-axis lattice parameter of 3.931 Å, in good agreement with the value reported in the literature.^38^ After confirming the crystallinity and the thickness of the films, we transfer individual flakes of SRO (6.3 nm) and STO (82 nm) on top of prepatterned SiO_2_/Si substrates with circular cavities with diameters from 3 μm to 10 μm and depths of 285 nm, using the vdW pick up technique.^39,40^

**Figure 2:**
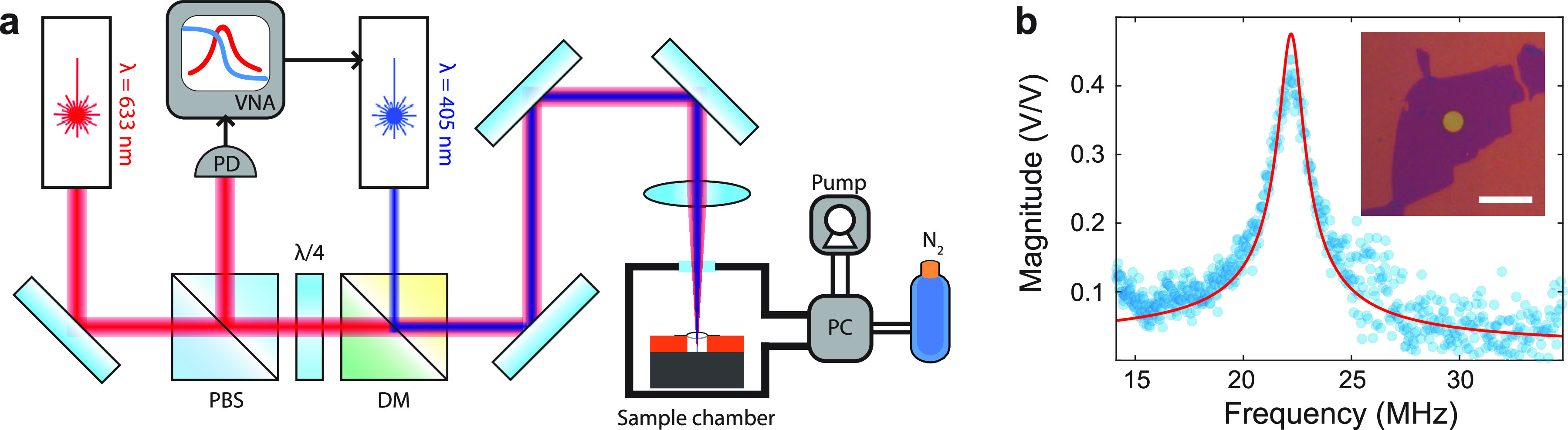
(a) Schematic illustration of the measurement setup. Vector network analyzer (VNA) sends an amplitude modulated signal to the blue laser diode which optothermally actuates the membrane while the red He–Ne laser reads out its motion. The reflected red laser light is detected at the photodetector (PD) and the signal is collected by the VNA. The pressure inside the sample chamber is controlled by the pressure controller (PC) which is connected to a scroll pump and a pressurized N_2_ gas bottle. PBS, polarized beam splitter; DM, dichroic mirror. (b) An example of a resonance peak of a SRO (16 u.c.) device with a harmonic oscillator fit in red. Inset: optical image of the device. A SRO flake is stamped on top of a circular cavity in SiO_2_/Si. Scale bar is 10 μm.

Once the fabrication of suspended complex oxide membranes are completed, we measure the pressure dependence of the resonance frequencies using a laser interferometry technique as illustrated in Figure 2a. An intensity modulated blue (λ = 405 nm) laser excites the motion of the membrane which is situated in a pressure controlled environment. A continuous red laser (λ = 633 nm) monitors the movement of the membrane. The reflected signal is collected by a photodetector (PD) and the signal is sent to a vector network analyzer (VNA). Figure 2b shows an example resonance peak of a SRO flake suspended over a circular SiO_2_/Si cavity (see inset). A harmonic oscillator function is fitted to the data (red) which is used to extract the resonance frequency as a function of sample chamber (SC) pressure.

**Figure 3:**
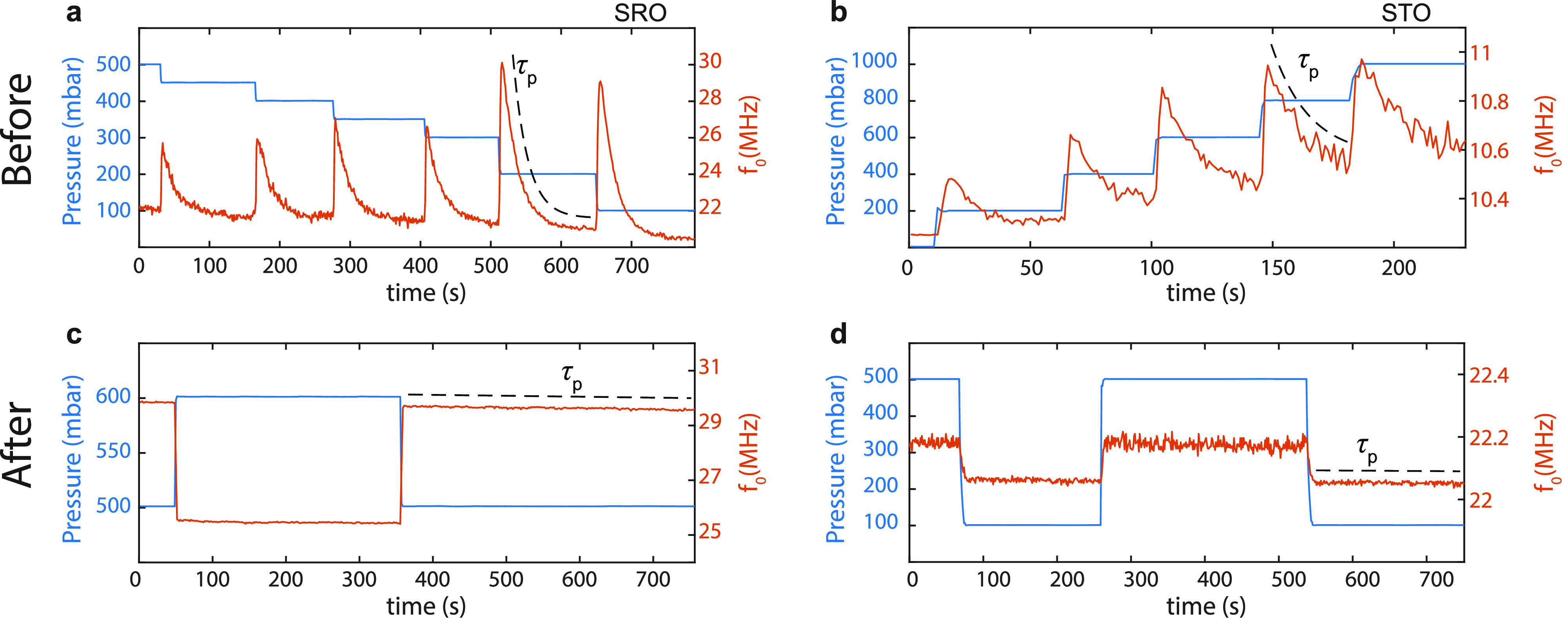
Pressure response of the resonance frequency before (a,b) and after (c,d) annealing. Left column shows the behavior of a 16 unit cell (6.3 nm) SRO device and the right column shows the behavior of 82 nm STO device. The external pressure controlled by the pressure controller is plotted in blue on the left *y*-axes and the resonance frequency is plotted in orange on the right *y*-axes.

The time-dependent resonance frequencies of SRO and STO devices directly after transfer over the cavities are shown in Figure 3a,b. The SC pressures are adjusted in a stepwise fashion while the frequency is swept to capture the resonance peak. After fitting the data to a harmonic oscillator function, the resonance frequency is extracted and plotted (orange, right *y*-axis). Before the sealing procedure, after the pressure changes both SRO and STO membranes show sudden increases in the resonance frequencies followed by exponential decays of time constant τ_p_. This behavior suggests that the membranes are tensioned due to the change in the pressure difference in and outside of the cavity, which then quickly equilibrates due to the permeation of gas molecules. In both SRO and STO, the average permeation time constants τ_p_ are approximately 21 and 14 s, respectively (see Supporting Information Section S.IV for details on the analysis).

To reduce the gas leakage, a self-sealing procedure is performed in which the samples are annealed in air at atmospheric pressure at elevated temperature (for 1 h at 300 °C for SRO and for 15 min at 400 °C for STO). These are parameters similar to those used in DWB of LiNbO_3_.^18^ After this procedure, the measurements from Figure 3a,b are repeated for both SRO and STO samples and shown in Figure 3c,d, respectively. The sudden spike in the resonance frequency followed by a fast decay is not observed in Figure 3c, but instead a slow reduction in the resonance frequency is observed. By fitting an exponential decay to the slow reduction in the resonance frequency, we find a τ_p_ of 1.1 × 10^4^ s. Similar behavior is observed in the STO device after annealing for 15 min at 400 °C. Before annealing, the mean permeation time constants of STO is τ_p_ = 13.9 s, which increases to 1.2 × 10^5^ s after a self-sealing procedure. As shown in Figure 3d, no observable decay in the resonance frequencies is present but there are small drifts. It is worth mentioning that due to the minute variations in the resonance frequency, fitting the data from Figure 3c,d to an exponential decay is difficult and results in large fit errors. However, we estimate a lower bound of 10^3^ s.

Next, the adhesion at the interface of complex oxides and the substrate is further tested using a picosecond ultrasonics method. An ultrafast optical pump–probe setup is used to generate and detect GHz acoustic waves in solids. This allows characterization of the adhesion between the thin layer and the substrate, since these waves are sensitive to the boundary conditions at the interface between two different materials.^41−43^ For example, this method is used to probe the adhesion properties of metal layers evaporated on glass surfaces^42^ or to characterize the adhesion of vdW materials.^44^

**Figure 4:**
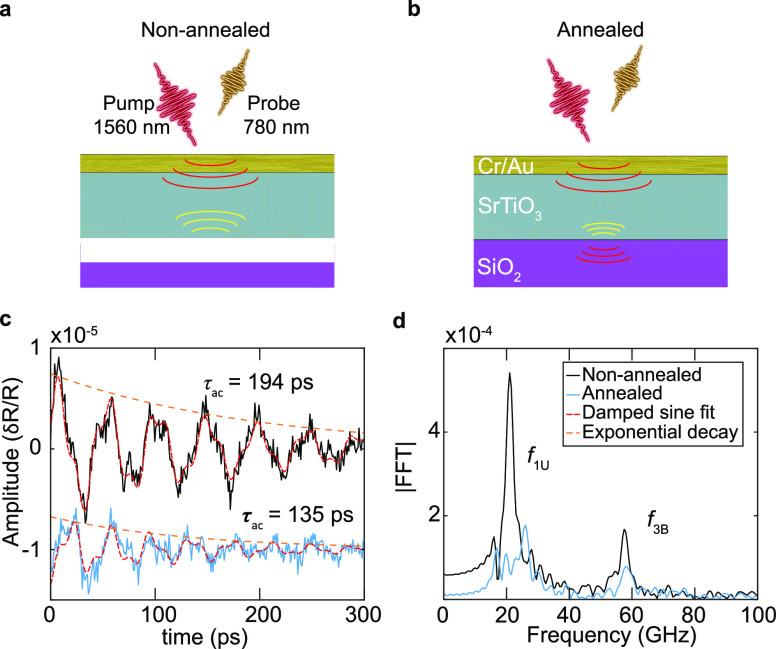
Cross sectional illustration of the pump–probe measurement in (a) nonannealed STO sample and (b) annealed STO sample. The red acoustic waves depict the propagating wave from the pump pulse and yellow from the reflection at the interface. A 33 nm thick metal layer is deposited on top for the ultrafast pump–probe measurements. (c) Examples of picosecond ultrasonics measurements on nonannealed (black) and annealed (light blue, offset in *y* for easier visualization) flakes of STO. Dashed red lines are fits to the damped sine function and the dashed orange lines depict the exponential decay envelopes. The *y*-axis shows the relative change in the optical reflection coefficient (δ*R*/*R*) of the probe pulse as a function of the time difference (*x*-axis) between pump and probe pulse. (d) Fourier transform of the waves in (c).

We prepare two sets of flakes on SiO_2_/Si transferred from the same batch of STO (thickness 82 nm). One set is untreated (Figure 4a) while the other is treated with the self-sealing procedure for 1 h at 400 °C (Figure 4b). Both substrates containing STO flakes are then coated with 33 nm of Au/Cr for optical pumping and probing (see Supporting Information Section S.XI for details). We use an asynchronous optical sampling (ASOPS) technique with a 1560 nm pump laser pulse and a 780 nm probe pulse laser to optothermomechanically generate and detect acoustic echos into individual STO flakes on time scales ranging from 1 ps to 10 ns. The duration of the pulses of both lasers is around 100 fs. Measurements are performed on four nonannealed and five annealed flakes. An example of the acoustic measurements on a nonannealed (black) and an annealed (light blue) STO flakes is presented in Figure 4c,d. The overall results are reported in Table 1.

**Table 1:** Results of Picosecond Ultrasonics Measurements on Four Nonannealed and Five Annealed STO Samples

	τ_ac_ (ps)	|*R*_ac_|	*K*_L_ (10^18^ N/m^3^)
nonannealed (4 flakes)	219.9 ± 50.0	0.81 ± 0.04	1.33 ± 0.20
annealed (5 flakes)	113.6 ± 17.1	0.70 ± 0.04	2.30 ± 0.64
theoretical values	59 (perfect contact) *∞* (total debonding)	0.45 (perfect contact) 1 (total debonding)	>20 (perfect contact) <0.1 (total debonding)

The acoustic wave echos inside the Au/Cr/STO assembly are observed in the nonannealed and annealed cases in Figure 4c. The decay of the amplitudes of the measurements in the different flakes (Figure 4c, for example) are fitted with a damped sine-wave to obtain the time constant τ_ac_ of the envelope, which characterizes the decay rate of the waves due to reflections at the interface with the substrate, wherein acoustic energy is transmitted. This results in an average value of τ_ac_ = 220 ps for the nonannealed flakes and τ_ac_ = 114 ps for the annealed flakes (see Table 1). From these values of τ_ac_, we calculate the associated acoustic reflection coefficient^44^ |*R*_ac_|, at the STO/SiO_2_ interface. An average value of |*R*_ac_| = 0.81 is found for the nonannealed flakes and |*R*_ac_| = 0.70 for the annealed flakes. These reflection coefficients allow the calculation of their associated interfacial stiffnesses *K*_*L*_, which are a direct measure of the adhesion at the interface; a higher *K*_L_ corresponds to a stronger adhesion (see Supporting Information Section S.XI for more information). A value of *K*_L_ = 2.30 × 10^18^ N/m^3^ is found for the annealed flakes, which is larger than that of the nonannealed flakes, *K*_L_ = 1.33 × 10^18^ N/m^3^. After annealing, the interfacial stiffness increases, resulting in a better transmission of the acoustic energy to the SiO_2_/Si substrate during the successive reflections of the acoustic waves inside the Au/Cr/STO assembly and therefore to a weaker reflection coefficient and a faster decay in amplitude.

Figure 4d shows the Fourier transform of the temporal data in Figure 4c revealing that they are composed of two distinct frequencies. The low frequency (∼22 GHz) corresponds to the first mode of standing waves in the Au/Cr/STO assembly unbound from the underlying SiO_2_, calculated theoretically (see Supporting Information Section S.XI for details) at *f*_1U_ = 21 GHz. The higher frequency (∼57 GHz) likely corresponds to the third mode of the standing waves in the bounded assembly (calculated *f*_3B_ = 53 GHz). The difference between the theoretical values and the experimental ones could be caused by small variations in the thickness of the Au/Cr/STO assembly and by the value of the longitudinal sound velocities used to calculate these frequencies taken from the literature (see Supporting Information Section S.XI). In both samples, the presence of frequencies corresponding to unbounded (*f*_1U_) and bounded (*f*_3B_) cases shows that the adhesion here is intermediate^44^ (between perfect contact and total debonding). However, the amplitude of the components in the annealed case are weaker than in the nonannealed case (Figure 4d), since their attenuation by transmission of the acoustic energy to the substrate through multiple reflections in the Au/Cr/STO assembly is higher (difference in τ_ac_, Figure 4c). The increased adhesion found from these ultrafast picosecond ultrasonics measurements is consistent with the increased hermeticity observed in Figure 3.

**Figure 5:**
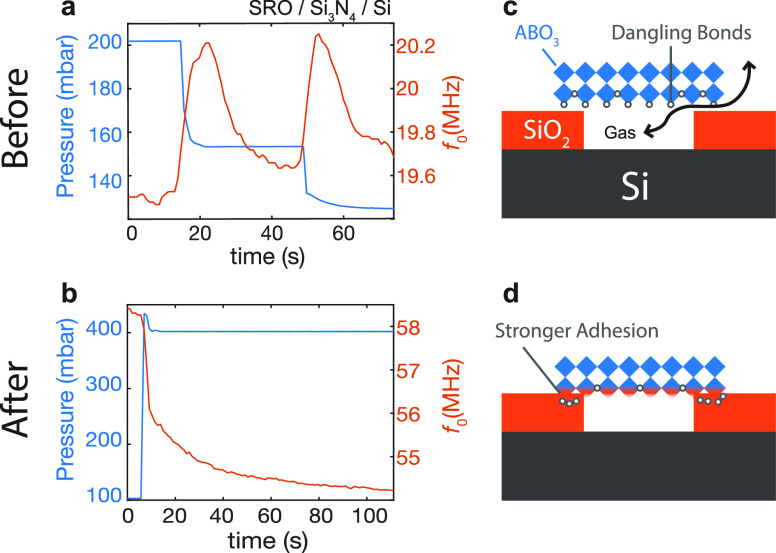
Pressure response (left *y*-axis, blue) of mechanical resonance (right *y*-axis, orange) in a SRO device fabricated on 350 nm Si_3_N_4_/Si (a) before annealing and (b) after annealing. Possible mechanism of the bonding is illustrated in panels c and d. (c) Before annealing, there are dangling bonds at the bottom of the flakes. The vdW gap between the SRO and the SiO_2_ allows for gases to pass through. (d) After annealing, vacancies bond with the oxygen in the substrate leading to a stronger bond to form at the interface.

Having used mechanical resonance measurements and picosecond ultrasonics measurements to establish the enhancement of the adhesion at the interface of complex oxides and SiO_2_, we now move on to investigate whether the observed adhesion increase is unique to the SiO_2_ substrate. For this purpose, we performed additional permeation measurements on nominally identical samples made on an oxygen-free substrate, Si_3_N_4_/Si. Figure 5a,b shows the time dependence of the resonance frequency of a SRO flake suspended over a cavity etched in Si_3_N_4_/Si. Figure 5a is taken before annealing and Figure 5b is taken after annealing for 1 h at 400 °C. Before annealing, the permeation time constant is τ_p_ = 6.02 s and after annealing for 1 h, it increases to τ_p_ = 22.5 s. Although a factor of 3.7 improvement is observed, the absolute leakage time constant after annealing in Si_3_N_4_ devices are on the order of those in SiO_2_ devices even before annealing (see Supporting Information Section S.V for the analysis on τ_p_). Investigation by means of energy dispersive X-ray spectroscopy (EDX) ruled out any role of SAO residues in the adhesion (see Supporting Information Section S.VI). The absence of the Al peak in the EDX spectra strongly suggests that SAO is indeed fully removed by water. Because of the above reasons, we hypothesize that the improved bonding is mediated by the presence of oxygen in the SiO_2_ substrate. A possible scenario is illustrated in Figure 5c,d where the adhesion is enhanced by the reaction between dangling bonds at the bottom of the complex oxide flake and the oxygen rich substrate at elevated temperatures.

Furthermore, it is worth to note the longevity of the improved adhesion on top of the SiO_2_ substrate. We have performed pick-up techniques widely used in the fabrication of vdW heterostructures using both polypropylene carbonate (PPC) and polycarbonate (PC), the latter of which has stronger adhesive properties and is thus more suitable for monolayer transfer.^39,45,46^ Nonannealed samples of SRO on SiO_2_ were easily picked up using both PPC and PC, while the samples annealed 8 months ago and stored in ambient conditions could not be removed from the substrate (see Supporting Information Section S.VIII). Unlike vdW materials which are able to detach from the substrate after annealing and subsequently storing in ambient conditions, the improvement in the adhesion seems to be longer-lasting in annealed oxides.

Although graphene and its family of vdW materials have demonstrated superior pressure sensing capabilities^1^ compared to the state-of-the-art made from Si,^6^ the leakage through the vdW gap between the material and the substrate still remains a key challenge to overcome. High hermeticities were obtained in exfoliated single layer graphene membranes in references,^9,47^ which would make them a great candidate for future sensing devices if they can be produced with high yield and reproducibility. Previous works have shown that the hermeticity may be improved by, for example, ironing with a diamond atomic force microscopy (AFM) tip^12^ or electron-beam induced deposition of SiO_2_.^11^ Unfortunately, neither methods are scalable, since they are too slow to apply over large areas. Furthermore, if only the edge of the flake is sealed and there is a puncture in a sealed flake, then all of the cavities underneath the flake are effectively vented. The intrinsic vdW nature and the difficulty in producing pinhole free 2D materials are the hurdles in fabricating reliable hermetically sealed vdW pressure sensors.

An alternative method which had been successfully used to measure the pressure changes in vented pressure sensors is through the squeeze-film effect, where the gases trapped between the membrane and the back-plate give rise to an added stiffness to the membrane thus increasing the resonance frequency. The responsivities in graphene squeeze-film pressure sensors (up to 9 kHz/mbar)^2^ already outperform that of the state-of-the-art Si-based sensors (200 Hz/mbar).^48^ The self-sealed complex oxide resonators presented in this work also outperform the Si-based squeeze film pressure sensor. The estimate of the responsivities extracted from Figure 3c,d are 42 ± 3 kHz/mbar and 296 ± 5 Hz/mbar for SRO and STO devices respectively. In particular, the sealed SRO device also outperforms the responsivities of graphene squeeze-film pressure sensor.^2^

The annealing procedure performed on the single crystal complex oxide flakes on SiO_2_ improves the hermeticity of the cavity as measured by mechanics and improves mechanical contact as measured by picosecond ultrasonics. As observed in the AFM topography of an SRO membrane before and after annealing, the thermal treatment seems to have caused a reduction in buckling resulting in a higher tension (see Supporting Information Section S.IX). This is likely due to the large mismatch in the thermal expansion coefficients of SRO^49^ and Si^50^ which causes the tension to increase when cooling down after clamping at higher temperatures. Furthermore, the PC pick-up technique widely used in the fabrication of the vdW heterostructures is ineffective in removing the annealed flakes from SiO_2_ even after prolonged storage in ambient conditions. Therefore we conclude that after the heat-activated self-sealing procedure, the adhesion is better and contact between the PLD layer and the substrate is more intimate. This increase may be caused by the removal of water and by formation of chemical bonds. Both mechanisms might play a role. However, the permeation time constant does not seem to increase by comparable magnitudes in samples created on Si_3_N_4_/Si substrates. This seems to suggest that chemical bond formation is the most likely, as water removal will likely happen for both substrates. This mechanism is similar to direct wafer-to-wafer bonding techniques that are in widespread use in the semiconductor industry.^18,51−55^ As reported by Weldon et al., water trapped between wafers dissociates at high temperatures and creates additional oxide layers, potentially aiding in the bonding process of Si wafers.^56^ Also, the bonding may be influenced by the surface chemistry^57^ and morphology^58^ of the film and the substrate, in which case the implementation of well-established pretreatment protocols used in wafer bonding technologies such as polishing,^26^ chemical treatments,^59^ or plasma treatments^60^ may be able to improve the adhesion of complex oxides on SiO_2_ further.

Thanks to the advent of the water-releasing technique,^27^ it is possible to synthesize free-standing single-crystal complex oxides and transfer them onto cavities for MEMS applications as presented in this work. One key difference between vdW materials and free-standing complex oxides is the existence of interlayer covalent bonds. We propose that by controlling the concentration of oxygen vacancies in the complex oxide, the density of dangling bonds at the surface may be tuned. However, care should be taken during the PLD growth of complex oxides since the presence of crystal defects has been shown to allow the permeation of small molecules such as water.^61^ Therefore, we expect to be able to further improve the adhesion, and thus the hermeticity, by optimizing the defect density and annealing conditions.

In summary, we have investigated the use of free-standing complex oxides SrRuO_3_ and SrTiO_3_ for pressure sensing applications and presented a self-sealing method based on annealing to improve the hermeticity. Gases permeate along the vdW–substrate interface, and the elimination of this leakage path is a key toward fabricating next-generation pressure sensors. We realized a leap toward this goal by promoting stronger adhesion to form at the interface of complex oxides and SiO_2_. Improvements in the gas permeation time constant as well as the contrast in the acoustic impedance at the interface suggest that the interface adhesion of complex oxides (SrRuO_3_ and SrTiO_3_), and SiO_2_ is stronger after annealing. We further investigated the effect of the substrate on the interfacial adhesion by performing the permeation measurements on devices made on Si_3_N_4_/Si. Since significant improvements in time constant are only observed on SiO_2_ substrates, it is likely that the oxygen atoms on the SiO_2_ substrate surface play an important role in mediating the adhesion. We anticipate future fundamental studies of complex oxide heterostructures, which are formed by annealing a van der Waals heterostructure of individual flakes that form chemical bonds between the layers upon annealing. Our work presents a first step toward implementing free-standing complex oxides as an alternative to silicon and 2D materials in next generation MEMS and NEMS sensors.

## Supporting Information Available

Methods, in situ RHEED data during PLD growth, XRD and AFM characterization of free-standing STO, permeation time constants of SRO and STO on SiO_2_, permeation time constants of SRO on Si_3_N_4_, EDX of free-standing SRO, AFM of substrate after water etching, optical images, and AFM of flakes not being picked up by a polymer, AFM of flakes before and after annealing, AFM of SRO flake ruptured due to large pressure difference (see also TOC graphic), ultrafast acoustics (methods, results simulations, and analysis) (PDF)
