# Supplementary material for: Self-Sealing Complex Oxide Resonators
Source: Nano Lett. 2022 Feb 4;22(4):1475–82. doi: 10.1021/acs.nanolett.1c03498 (PMC8880390; doi:10.1021/acs.nanolett.1c03498)
Supplement: Supplementary file 1 — nl1c03498_si_001.pdf [file nl1c03498_si_001.pdf]

Supplementary Information for:

Self-sealing complex oxide resonators

Martin Lee,[1] Martin P. Robin,[2] Ruben H. Guis,[2] Ulderico Filippozzi,[1] Dong Hoon Shin,[1] Thierry C. van Thiel,[1] Stijn P. Paardekooper,[2] Johannes R. Renshof,[1] Herre S. J. van der Zant,[1] Andrea D. Caviglia,[1] Gerard J. Verbiest,[2] and Peter G. Steeneken[1, 2]

[1])*Kavli Institute of Nanoscience, Delft University of Technology, Lorentzweg 1, 2628 CJ Delft, The Netherlands.*

[2])*Department of Precision and Microsystems Engineering, Delft University of Technology, Mekelweg 2, 2628 CD Delft, The Netherlands.*

S.I. METHODS

Pulsed laser deposition of SRO/SAO/STO

The SRO and SAO films are grown by pulsed laser deposition on TiO_2 termiated STO(001) substrates purchased from CrysTec GmbH. Pulses of KrF excimer laser are delivered at 1 Hz at fluences of 1.7 J/cm^2. SAO is grown at 800 $°C$ and 2×10^{-6} mbar pressure and SRO at 550 $°C$ and 1×10^{-1} mbar O_2 pressure. After growth, the stack is annealed at 500 $°C$ for an hour and cooled down to room temperature in 300 mbar O_2.

Pulsed laser deposition of STO/SAO/STO

The freestanding STO film is fabricated in a similar manner to the SRO. STO is grown in 1×10^{-6} mbar O_2 pressure at 800 $°C$. The excimer laser is delivered at 1 Hz at a fluence of 1.2 J/cm^2. After growth, the stack is annealed at 500 $°C$ for an hour and cooled down to room temperature in 300 mbar O_2.

Release and Transfer onto bare SiO_2/Si

Prior to releasing from the growth substrate, a commercial polydimethylsiloxane (PDMS) film (Gel-Pak ®) is attached to the surface of the SRO/SAO/STO stack. The stack covered by PDMS is then submerged under deionized water for 24 hours. Once the SAO is fully etched away and only SRO remains on the PDMS, the substrate detaches from the PDMS. The SRO/PDMS stamp is removed from water, dried in with N_2 gas and is ready to stamp. The flakes are then transferred onto a bare SiO_2/Si chip using the viscoelastic stamping technique[1]. This dummy SiO_2/Si chip is used as an intermediate substrate to place down the flakes for XRD characterization. From this dummy chip, flakes of adequate size are chosen for the final transfer using an optical microscope.

Polymer assisted pick up technique

The polymer assisted pickup technique using polypropylene carbonate (PPC)[2] coated PDMS dome[3] is used to transfer the chosen flake from the dummy SiO_2/Si to the device chip. The PPC covered PDMS dome is brought in contact with the chosen flake and the chip is heated beyond the

T_g ~ 45 °C. Once PPC is molded into the shape of the flake, the stack is cooled down to room temperature and the dome along with the flake is detached from the SiO_2/Si. The flake is then transferred onto a prepatterned SiO_2/Si device consisting cavities etched into SiO_2/Si. Finally the flake is released above 60 °C leaving only the flake.

Prepatterend SiO_2/Si

Dry thermal oxide of 285 nm, grown on highly doped (Si++) silicon is used as the substrate. Vistec EBPG 5000+ is used to expose the cavity defined in 500 nm of AR-P 6200 positive e-beam resist. After exposure and development, the cavities are dry etched into the SiO_2/Si using CHF_3 and Ar plasma until all the SiO_2 is removed. Remainder of AR-P 6200 is removed in PRS-3000 over night, rinsed and blow-dried. The substrates are further cleaned in O_2 plasma asher for 3 minutes. Similar procedure is employed for Si_3N_4/Si chips with 350 nm LPCVD grown Si_3N_4.

Annealing

The annealing takes place in ambient conditions on top of a VWR hot plate above 300 °C for 15 minutes to 1 hr.

Samples for ultra-fast acoustics

For the measurements of the acoustic boundary conditions using ultra-fast pump-probe method, separate samples need to be fabricated with metal layers deposited on top of the flakes. The metal layer is necessary to reflect the probe and absorb the pump to generate an acoustic pulse. Two dummy SiO_2/Si are prepared with several flakes of 82 nm STO. On both chips, 3 nm of Cr and 30 nm of Au are deposited using a Temescal e-beam evaporator.

S.II. RHEED

Reflection high energy electron diffraction (RHEED) is used to monitor the growth of the 82 nm STO used in the main text. In Fig. S1a-c, RHEED images of the substrate, SAO buffer layer and STO (82 nm) are shown. The RHEED intensities of the side spots/streaks are used to monitor the layer-by-layer growth of SAO and STO as shown in Fig. S1d.

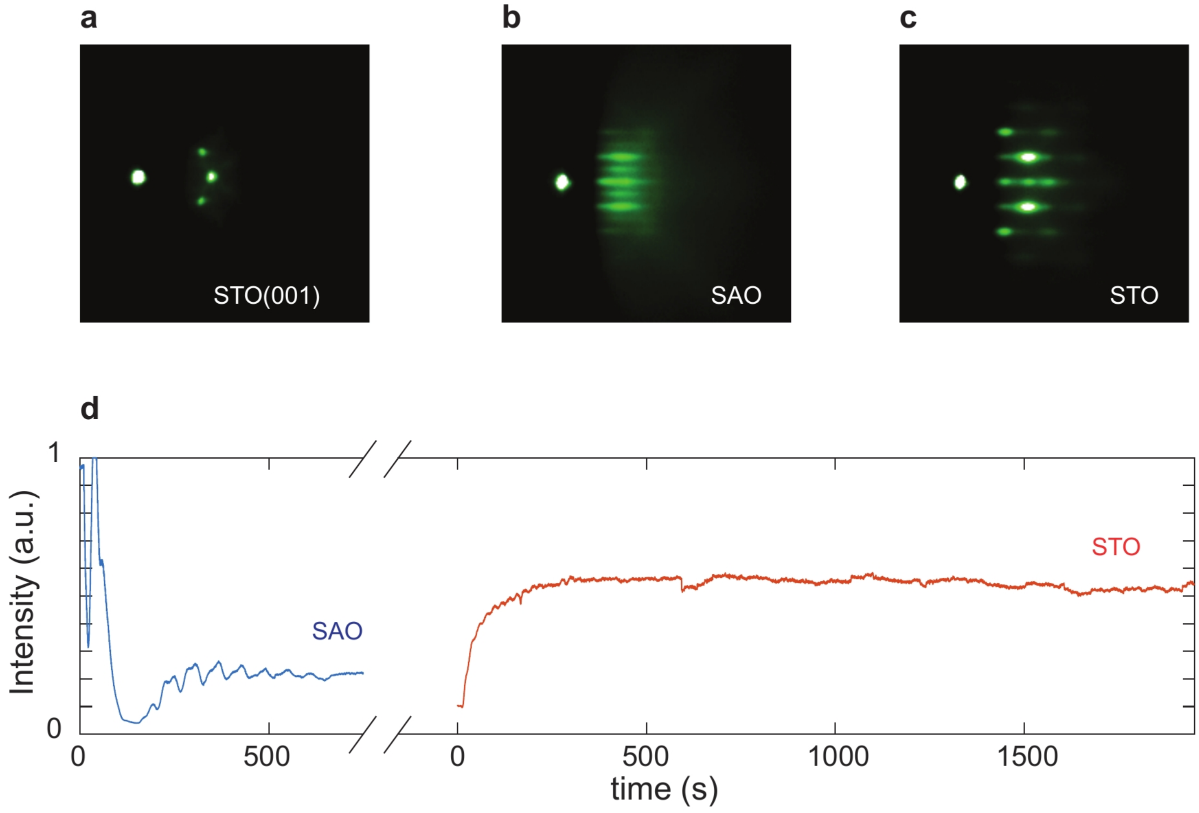

FIG. S1. Reflection high energy electron diffraction images of **a** the STO(001) substrate, **b** SAO buffer layer on STO(001), and **c** STO on SAO/STO(001). **d** RHEED oscillations of SAO and STO monitored during growth

S.III. CHARACTERIZATION OF STO

Figure S2a shows a X-ray refractometry (XRR) performed on a 218 nm thick STO stamped on SiO_2/Si and Figure S2b shows a X-ray diffraction (XRD) of the same film. The XRR shows multiple oscillations occuring between 1 ° and 2 ° indicating the uniformity of the film. Fast fourier transform analysis of this film gives a peak near 85 nm which agrees well with the atomic force microscopy (AFM) data shown in Fig. S3

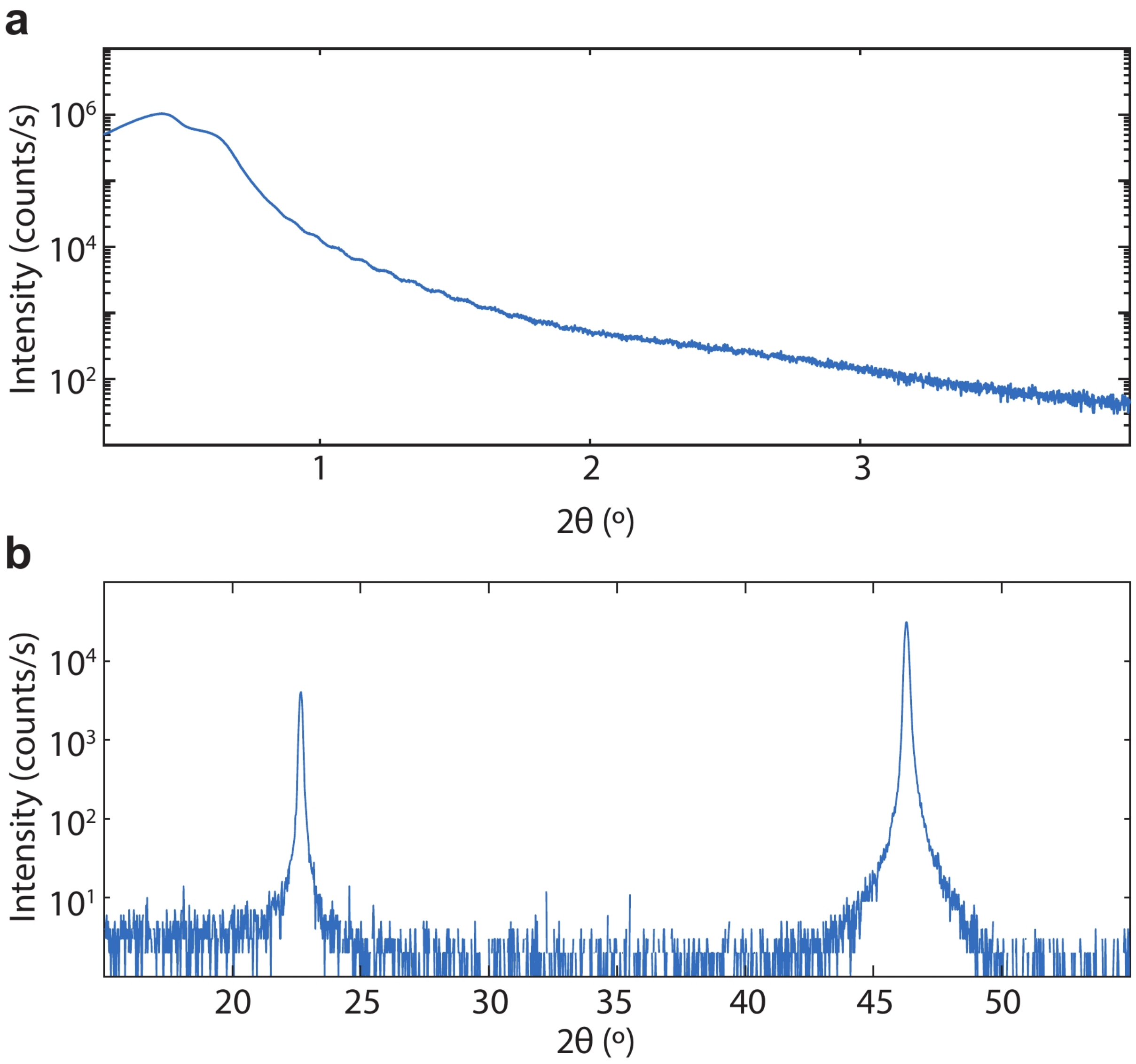

FIG. S2. **a** X-ray refractometry (XRR) performed on free-standing STO stamped on SiO_2/Si. **b** X-ray diffraction (XRD) of the same film showing STO (001) and STO (002) peaks.

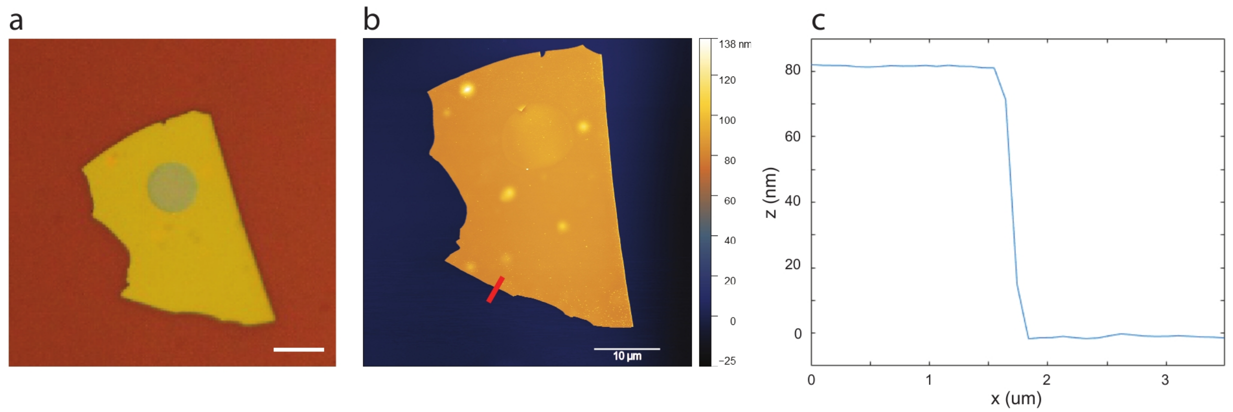

FIG. S3. **a** Optical image of a STO flake suspended over a 10 µm cavity. Scalebar: 10 µm. **b** Atomic force microscopy (AFM) image of the device. Red line indicates where a profile is taken. **c** Profile at the edge of the flake as indicated by the red line in **b**. Thickness of 82 nm is measured.

S.IV. PERMEATION TIME CONSTANTS

SRO device

Figure S4 show the pressure response of the resonance frequency before and after annealing. Before annealing, an average permeation time constant of τ_p = 20.75 seconds is extracted. Individual τ_p are listed in table S1. After annealing, this increases to $\tau_p = 1.1\times10^4$ seconds.

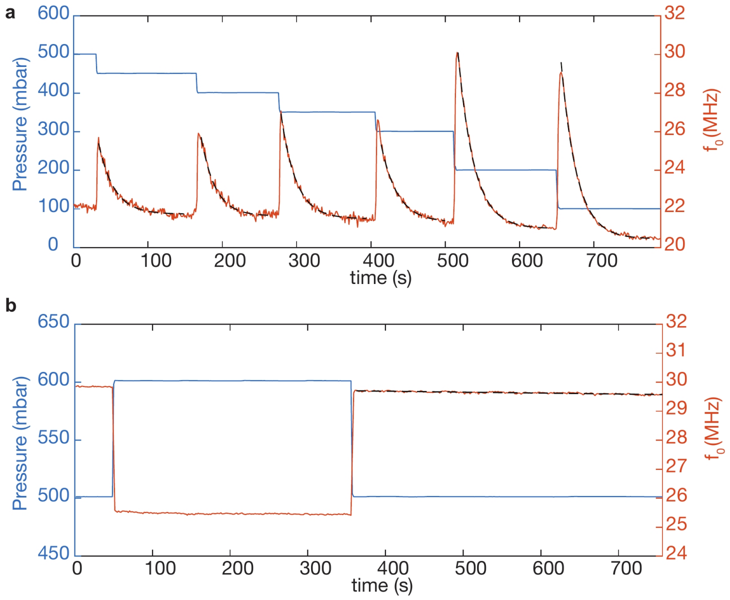

FIG. S4. Pressure (left y-axis, blue) response of mechanical resonance (right y-axis, orange) of SRO **a** before annealing **b** and after annealing. Black dashed lines are the decay curves taken to extract the permeation time constant τ_p.

STO device

Figure S5 show the pressure response of the resonance frequency before and after annealing. Before annealing, an average permeation time constant of τ_p = 13.87 seconds is extracted. Individual τ_p are listed in table S2. After annealing, this increases to $\tau_p = 1.2\times10^5$ seconds.

	τ_p(s)
curve 1	20.79
curve 2	18.95
curve 3	20.14
curve 4	22.16
curve 5	21.45
curve 6	21.02
mean	20.75

TABLE S1. Permeation time constants of decay curves in Fig. S4a

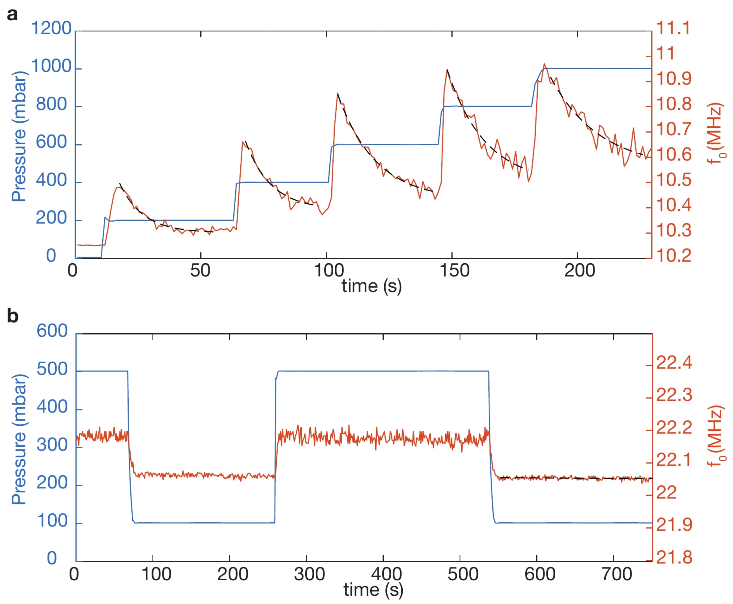

FIG. S5. Pressure (left y-axis, blue) response of mechanical resonance (right y-axis, orange) of STO **a** before annealing **b** and after annealing. Black dashed lines are the decay curves taken to extract the permeation time constant τ_p.

	τ_p(s)
curve 1	9.34
curve 2	10.16
curve 3	15.24
curve 4	15.17
curve 5	19.46
mean	13.87

TABLE S2. Permeation time constants of decay curves in Fig. S5

S.V. SRO ON SI_3N_4

Figure S6a&b are the pressure dependent resonance frequency data taken on a SRO flake stamped on Si_3N_4 substrate. A permeation time constant of τ_p = 6.02 s is extracted from the dashed black line in the data before annealing in Fig. S6a. Similarly, a τ_p = 22.5 s is extracted from the dashed black line in the data after annealing in Fig. S6b.

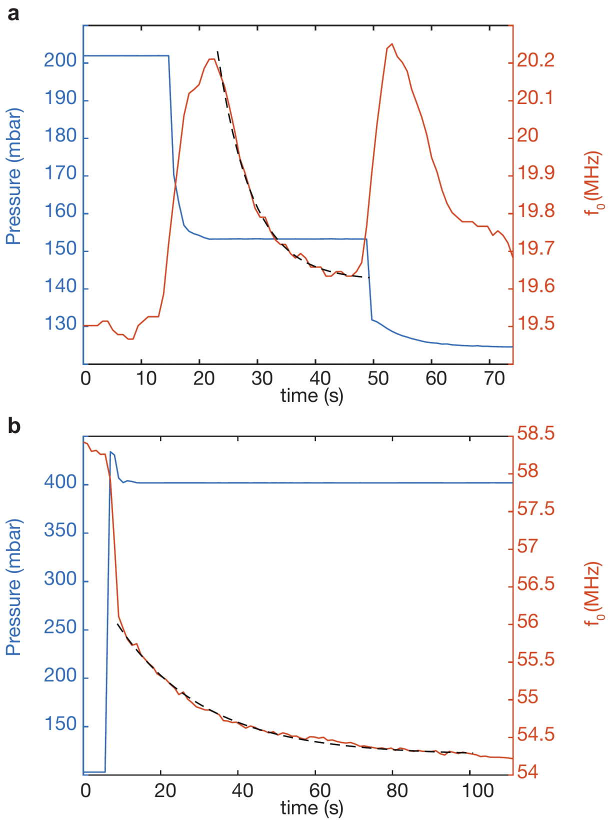

FIG. S6. Pressure response (left y-axis, blue) of mechanical resonance (right y-axis, orange) in a SRO device fabricated on 350 nm Si_3N_4/Si **a** before annealing, and **b** after annealing. Dashed black lines are exponential fits to extract the τ_p. Before annealing, τ_p = 6.02 s and after annealing τ_p = 22.5 s.

S.VI. EDX OF FREE-STANDING SRO

It is worth noting that the atomic force microscopy (AFM) profile of SRO flakes on SiO_2/Si are 10.9 nm while the XRD data shown in the main text suggests a thickness of 6.3 nm. The discrepancy in the measured thicknesses between the two methods can be attributed to a few things. First, there could be an overestimation of the thickness from AFM measurements due to tenting effect, water layer, or dirt. Shearer *et al.* have shown that the van der Waals gap between a flake and the substrate can often give a gross overestimation on the thickness of the flake when measured with an AFM[4]. Second, SAO may not have fully etched away, leaving behind some pillars of residues which also can attribute to the tenting effect. It is possible to hypothesize that the bonding is mediated by the residual SAO layer where the half etched SAO residues provide dangling bonds which could readily bind with the SiO_2 underneath. In order to eliminate the hypothesis that this bonding is mediated by the remnant SAO layers providing reactive dangling bonds underneath the SRO and STO flakes, we have performed energy dispersive X-ray spectroscopy (EDX) of the flakes and AFM topography analysis of the STO substrate after the release of the layers. Due to the absence of the Al peak in the EDX as well as the stepping terraces observed in the AFM of the substrate (next section), we can safely rule out the hypothesis that there is a remnant SAO residue which bonds to the SiO_2.

Figure S7a shows a scanning electron microscopy image (SEM) of the SRO flakes stamped on SiO_2/Si. An EDX spectrum is taken in one of the flakes indicated by a marker in Fig. S7a. The EDX spectrum plotted in Fig. S7b shows the elemental analysis which includes Si, O, Sr and Ru. These arise from both the flake and the substrate. However, there is no peak at 1.486 keV (green dashed line) which corresponds to the energy of Al. This indicates that the sacrificial water soluble SAO layers underneath the films of interest (SRO and STO) are fully etched away. SEM and EDX are performed using FEI Helios G4 CX at 5 keV and at a tilt of 52 degrees.

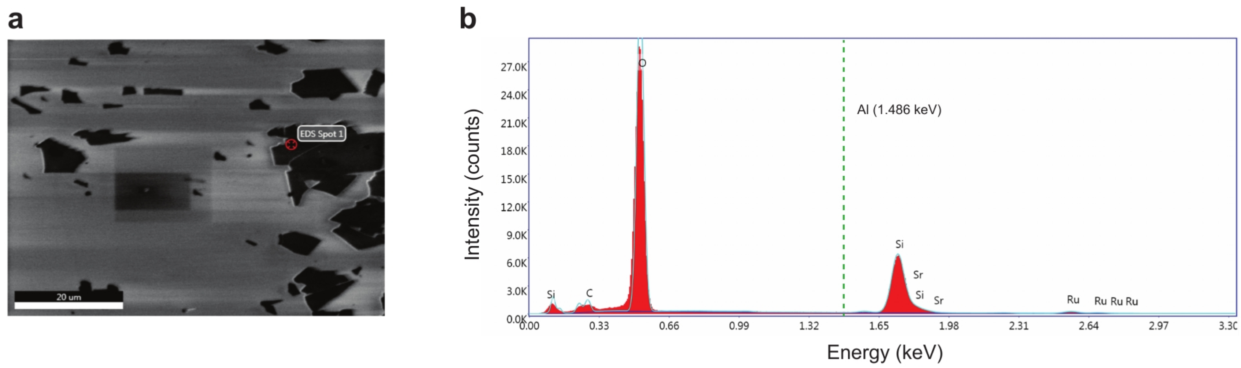

FIG. S7. **a** Scanning electron microscopy image of free-standing SRO stamped on SiO_2/Si taken at 52 degree tilt . The spot where energy dispersive X-ray spectroscopy (EDX) is performed is indicated with a marker. **b** EDX of the film showing contributions from Si, O, Sr and Ru but not Al (1.486 keV green dashed line) suggesting that $Sr_3Al_2O_6$ is fully etched away in water.

S.VII. AFM OF SUBSTRATE AFTER WATER ETCHING

Figure S8 shows an AFM image of the substrate after water etching. The bright left side still has SRO/SAO while the right side is the bare STO (001) substrate. Hints of terraces can still be seen on the bare STO after etching away the SAO, suggesting that most of the SAO is removed in water.

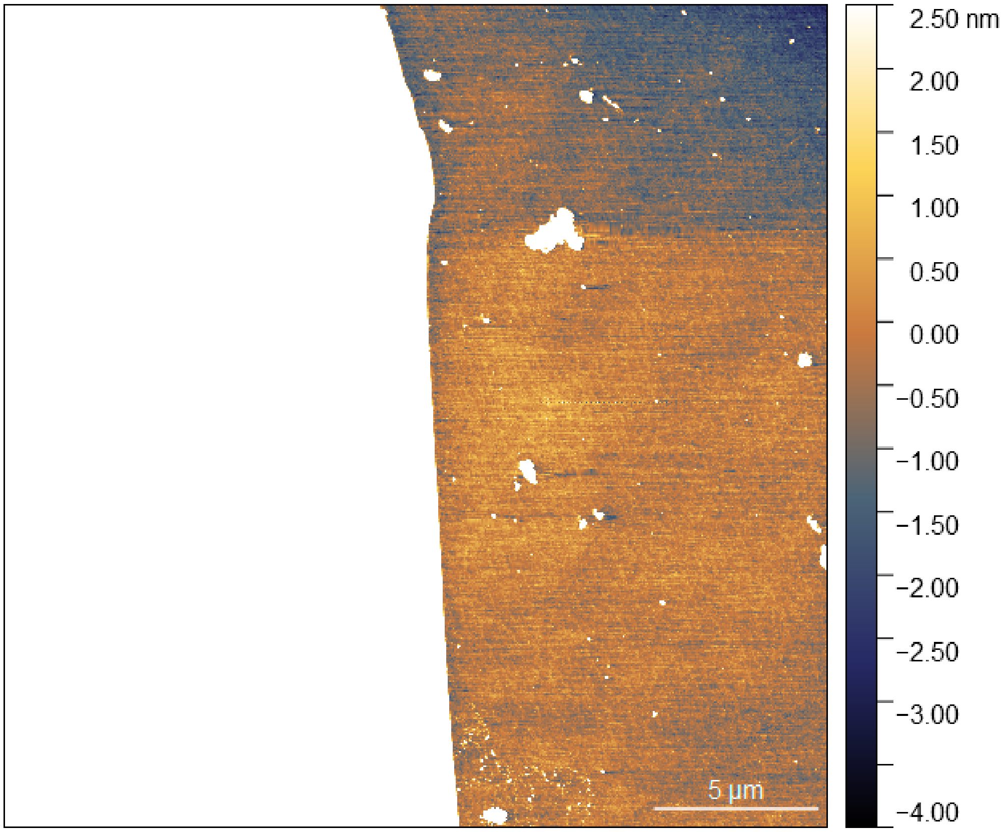

FIG. S8. AFM image of the substrate after water etching. Bright left side still has SRO/SAO on STO(001) and the right side is the bare STO(001) surface after SAO is etched away. Signs of terraces are visible.

S.VIII. OPTICAL IMAGES AND AFM OF FLAKES NOT BEING PICKED UP BY POLYMER

In this section, we provide an additional evidence for the improved adhesion of complex oxide flakes on SiO_2 upon annealing. In Figs. S9 and S10, we compare the maneuverability of non-annealed and annealed SRO flakes, respectively. In Fig. S9, SRO flake on a dummy SiO_2 is picked up using a standard polycarbonate/PDMS dome technique[3,5]. It can be observed that the non-annealed SRO flakes are easily picked up from the original dummy SiO_2 and transferred to a patterned SiO_2/Si. However, the same method used on annealed SRO flakes is unable to detach the flakes from the SiO_2/Si substrate. Figure S10 illustrates the process of the attempt to pick up annealed SRO flakes. In Fig. S10a, an AFM scan is shown of an area consisting of a flake stamped on top of a Pd electrode recessed into SiO_2/Si. In Fig. S10b-d, optical images of the pick up process attempt is shown. In Fig. S10d, it can already be seen that the SRO flake remains intact, seemingly unaffected by the transfer attempt. In Fig. S10e an AFM scan of the same area as Fig. S10a after the pick up attempt is shown. It shows that the flake is not damaged by the attempt. It is worth mentioning that we have not succeeded in picking up SRO from SiO_2 after annealing, but using a sharp needle in a probe station, we were able to scratch away SRO from SiO_2.

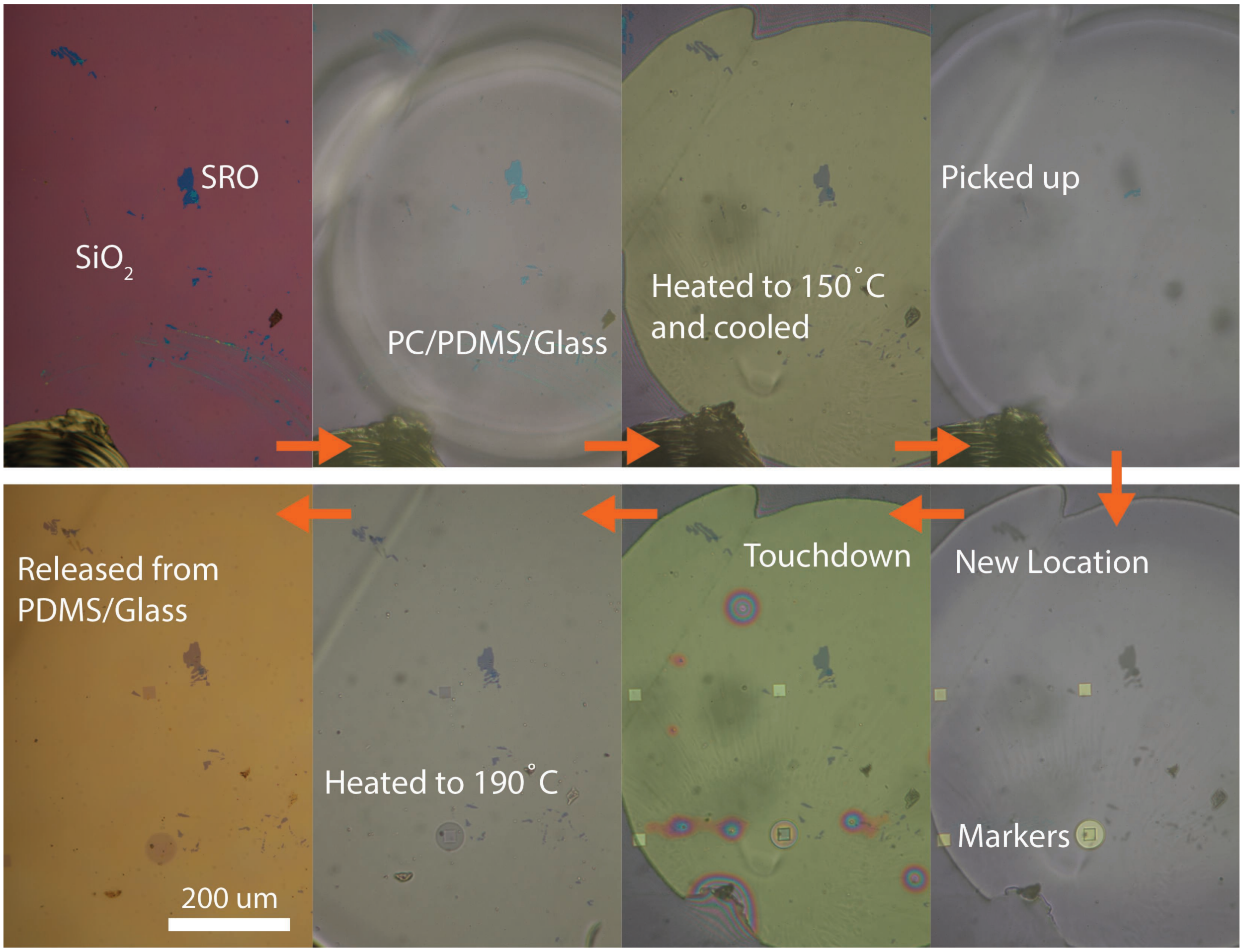

FIG. S9. Optical microscopy images of the flake pick-up process showing the transfer of non-annealed SRO from a dummy SiO_2/Si substrate to a patterned substrate. Polycarbonate is used as a sticky polymer to transfer the flakes (in this case, SRO).

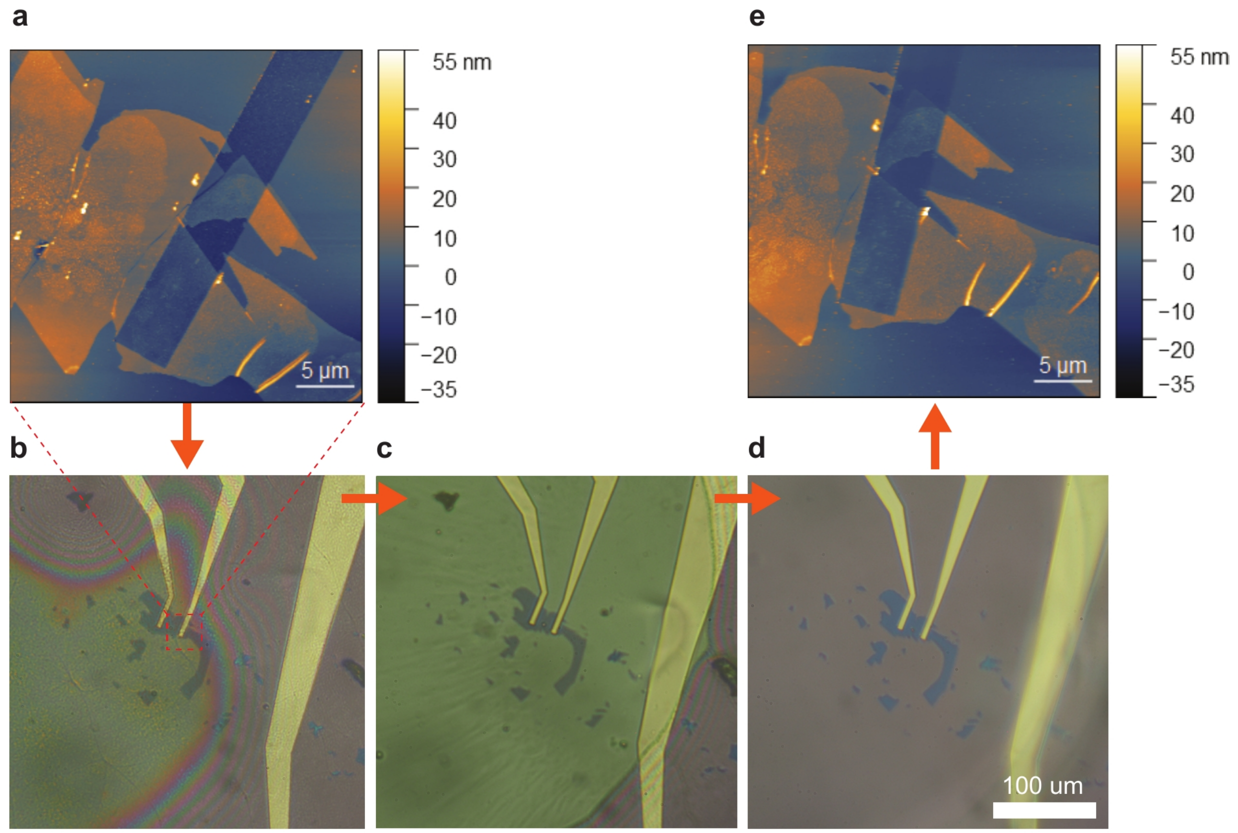

FIG. S10. AFM and optical microscopy images highlighting the inability to pick up the flakes of annealed SRO on SiO_2/Si. Polycarbonate is used as a sticky polymer to transfer the flakes. **a** AFM image of an annealed flake on top of a recessed electrode. **b-d** Optical images of the attempt to pick up the annealed flake using polycarbonate/ PDMS. **e** AFM image of the same same area after the pickup attempt.

S.IX. AFM OF FLAKES

In this section we use atomic force microscopy (AFM) to inspect SRO flakes before and after annealing. Figure S11a&b show the AFM images of SRO flakes across the edge of the flake (Fig. S11a) and on the drum (Fig. S11b). The line scan over the edge of the flake shows a step height of 14.2 nm (inset of Fig. S11a). Compared to the XRD data shown in the main text, which suggests a thickness of 6.29 nm there is an overestimation of the thickness by a factor of more than 2. This could be a result of the PPC residues as can be seen in both topography scans of Fig. S11a and b and an overestimation of the van der Waals gap due to water and/or dirt as described by Shearer *et al.* [4]. In Fig. S11c&d, AFM scans of the same device after annealing are shown. It can be seen in the inset of Fig. S11c that the line scan across the edge of the SRO flake is now 10.9 nm which is still an overestimate compared to the 6.29 nm we expect from the XRD data but is reduced from the data taken before annealing. Annealing can remove a large portion of the PPC residues. Also, a reduction in the amount of buckling is seen in Fig. S11d. Before annealing, the SRO membrane shows larger buckling amplitudes (Fig. S11b), likely arising from the PPC stamping process. However, after annealing the buckling height is largely reduced.

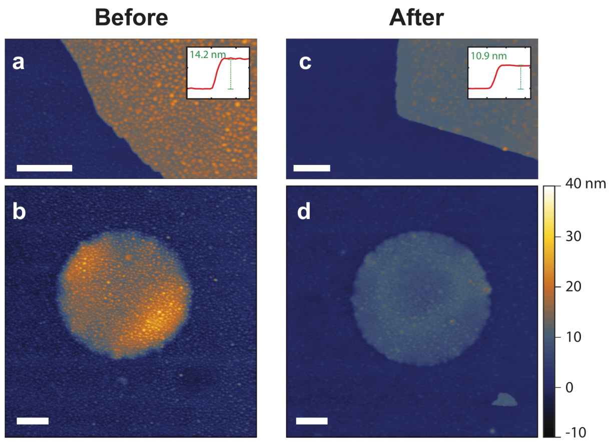

FIG. S11. **a** Atomic force microscopy (AFM) image of the edge of the SRO flake before annealing. The inset shows the step height of 14.2 nm. **b** AFM image of the suspended drum before annealing. **c** AFM image of the edge of the SRO flake after annealing. The inset shows the step height of 10.9 nm. **d** AFM image of the suspended drum after annealing.

S.X. EXPLODED DRUM

Figure S12 shows atomic force microscopy (AFM) images of a SRO flake on cavities of SiO_2/Si with pre-patterned electrodes. Shown device is annealed at 300°C for 15 minutes. After annealing the device is bonded and loaded into a Oxford He flow cryostat. Just prior to loading, the membrane is in a flat configuration as shown in Fig. S12a. In the process of loading, the membrane bulges up due to the pressure difference between the cavity, which is filled with $\sim$1 bar air and the cryostat which is pumped to $\sim$1 mbar before letting the He to flow. After the attempt to measure the electron transport in the cryogenic temperatures we removed the sample and observed the drum using AFM. As shown in Fig. S12b, the drum had been torn off from the device. We hypothesize that this has occurred in a violent fashion as illustrated in Fig. S12c. During the pumping phase, the membrane may have "exploded" from the device due to a sudden change in the pressure. Having sealed, the pressure difference between the cavity and the sample space in the cryostat will have had $\Delta P \sim 1$ bar.

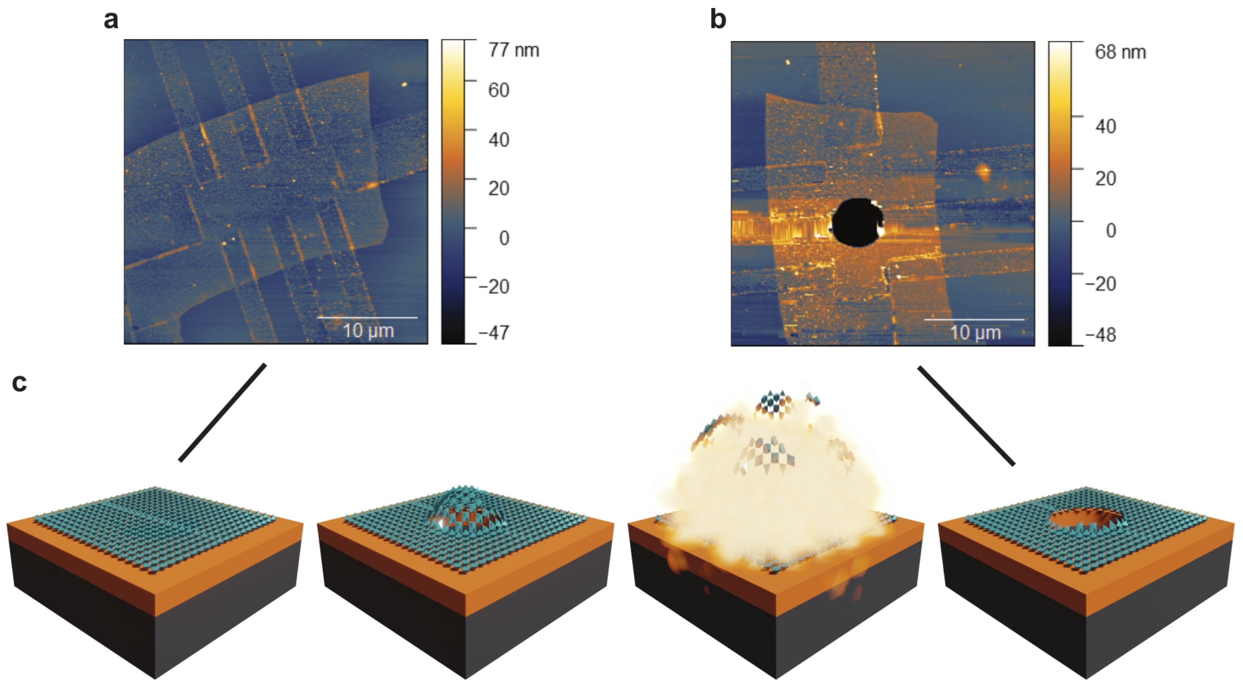

FIG. S12. Atomic force microscopy (AFM) images of an annealed SRO flake **a** before loading into a vacuum chamber and **b** after loading into a vacuum chamber. **c** Illustration of the explosion process in annealed SRO drum. In ambient conditions, SRO flake is in the flat configuration. When the sample chamber is abruptly pumped, the membrane bulges up due to the pressure difference between the cavity and the sample chamber. At base pressure of $\sim 10^{-3}$ bar, the membrane bursts leaving a SRO flake with a hole in the center.

S.XI. ULTRA-FAST ACOUSTICS

Method

Acoustic pulses in the GHz range are generated and detected using a picosecond ultrasonics technique[6]. By locally heating the sample with a 100 fs pump pulse, an acoustic pulse is generated. Using a probe pulse of the same duration, the acoustic reflections arriving back at the surface of the sample are measured. In this pump-probe experiment, the acoustic echoes are measured with the probe repetition rate at a slight offset from the 100 MHz pump, regulated by an asynchronous optical sampling (ASOPS) system from Menlo Systems. The pump and probe lasers are femtosecond erbium lasers (1560 nm), doubled in frequency (780 nm) for the probe. The average output power of the pump laser is $\sim$100 mW, and the one of the probe laser is set between 2 and 5 mW. Since the pump and probe repetition rates have a slight offset between them, at every next pulse the time of arrival between the pump and probe is slightly delayed in time. This way, the probe scans through the entire time window between 2 pump pulses (10 ns).

In the setup (Fig. S13), the probe beam is expanded to an appropriate size and passed through a half waveplate to achieve the correct polarization to be transmitted by the polarizing beam splitter. A quarter waveplate then shifts the initial linear polarization to an elliptical polarization. After passing through a dichroic mirror, where the pump path joins the probe path, the beams are focused on the sample through a sapphire plate, used for ultrasonics detection by means of conoscopic interferometry[7]. On the sample, the pump beam is absorbed to generate the acoustic waves. A part of the probe beam containing the acoustic signal is reflected. Then the quarter waveplate ensures that the reflected path is reflected towards the photodetector by the polarizing beam splitter. In this path, an iris diaphragm cuts out a part of the light, which is necessary for the conoscopic interferometry. A 250 MHz bandwidth, silicon based, amplified photodetector is used in this setup, allowing to detect each individual pulse.

To acquire the data we use a 600 MHz lock-in amplifier from Zurich Instruments with the additional Boxcar option. This option allows to combine a Periodic Waveform Analyser (PWA) and a Boxcar. Using the boxcar, the energy contained inside each detected probe pulses is extracted. From this, the PWA reconstructs the ASOPS signal by placing the pulses in the correct order. The sampled data are linked to the phase of an oscillator at the probe repetition rate. By plotting the sample amplitude against this phase, the laser pulses can be reconstructed (PWA). Using a gate

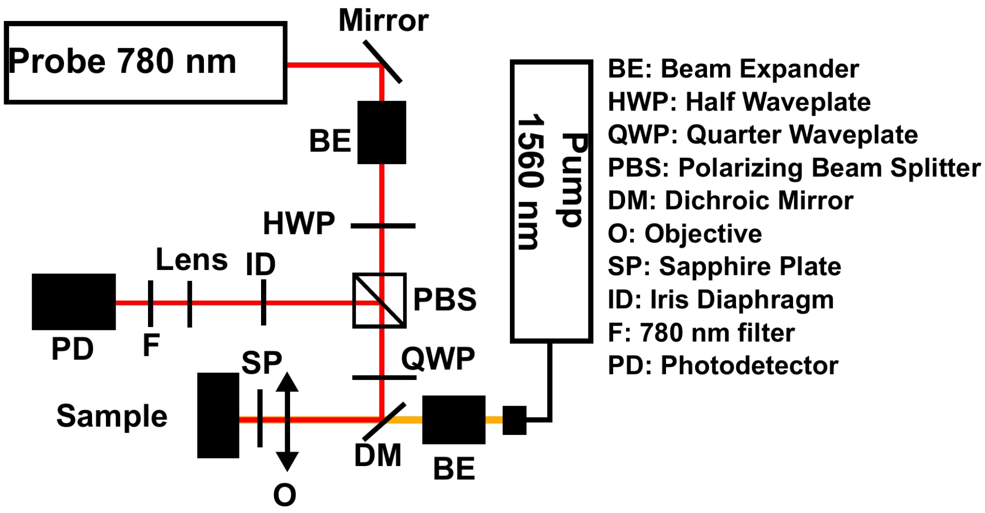

FIG. S13. Schematic illustration of the picosecond ultrasonics setup.

window this pulse can now be integrated and the amplitude can be derived (Boxcar). A second oscillator at the offset frequency between the pump and probe lasers, is also linked to the measurement. Using the phase of this second oscillator, the samples are divided in bins that are associated to corresponding time stamp within the measurement at which the sample was taken. By calculating the amplitude in each of this bins using the boxcar method and then concatenating them, the envelope signal of all the pulses is reconstructed.

Raw data

In the raw signal (Fig. S14), increases in the sample temperature caused by the pump laser pulses can be seen in the left and middle graph after a few ps. The effect is much higher in the non-annealed sample. This could be explained by the adhesion to the substrate. In the annealed sample, the improved adhesion facilitates a more efficient heat diffusion into the substrate, while in the non-annealed sample, it stays more confined inside the Au/Cr/$SrTiO_3$ assembly.

After $\sim$200 ps, a parasitic signal can be seen (also present without the pump excitation). This parasitic signal and the temperature increase are the origin of the high level component under $\sim$20 GHz in the spectra (Fig. S14 right). The part of the signal between the temperature increase and the parasitic signal ($\sim$50 ps – 200 ps) correspond to the acoustic signal, which is partially masked by the parasitic signal, as it can be seen in the non-annealed sample. The components of the spectra around $\sim$20 GHz and $\sim$57 GHz correspond to the frequency of these acoustic standing

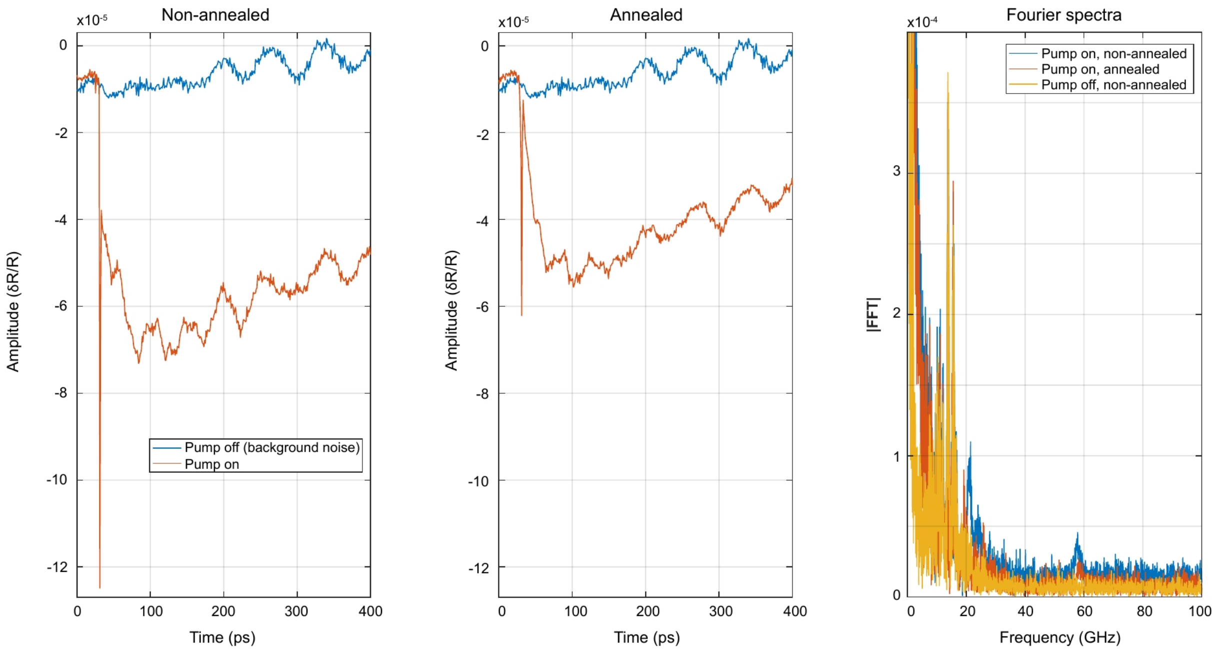

FIG. S14. Raw signal (zoom around the excitation). **Left:** Non-annealed sample. **Middle:** Annealed sample. **Right:** Fourier transformed spectra.

waves which are absent from the spectrum of the signal without excitation (Fig. S14 right, plotted in yellow) and are weaker in the annealed sample (Fig. S14 right, plotted in orange).

Filtered data

The signals are then high-pass filtered using a 18 GHz cut-off frequency to remove the parasitic background signals and the low frequency components of the temperature increase (Fig. S15). The acoustic waves become much more visible, as well their associated components in the Fourier spectra.

Fitting a sinusoidal envelope

Only the part of the signal corresponding to the acoustic waves is then considered. This one is firstly smoothed using a moving average filter on 5 samples and is then fitted using a minimisation algorithm to find the parameters of a damped sine as the following (Eq. S.1):

$$\text{fit}(t) = (A_1 \sin(2\pi f_1 t + \phi_1) + A_2 \sin(2\phi_2 t + \phi_2))e^{-t/\tau_{ac}}, \qquad \text{(S.1)}$$

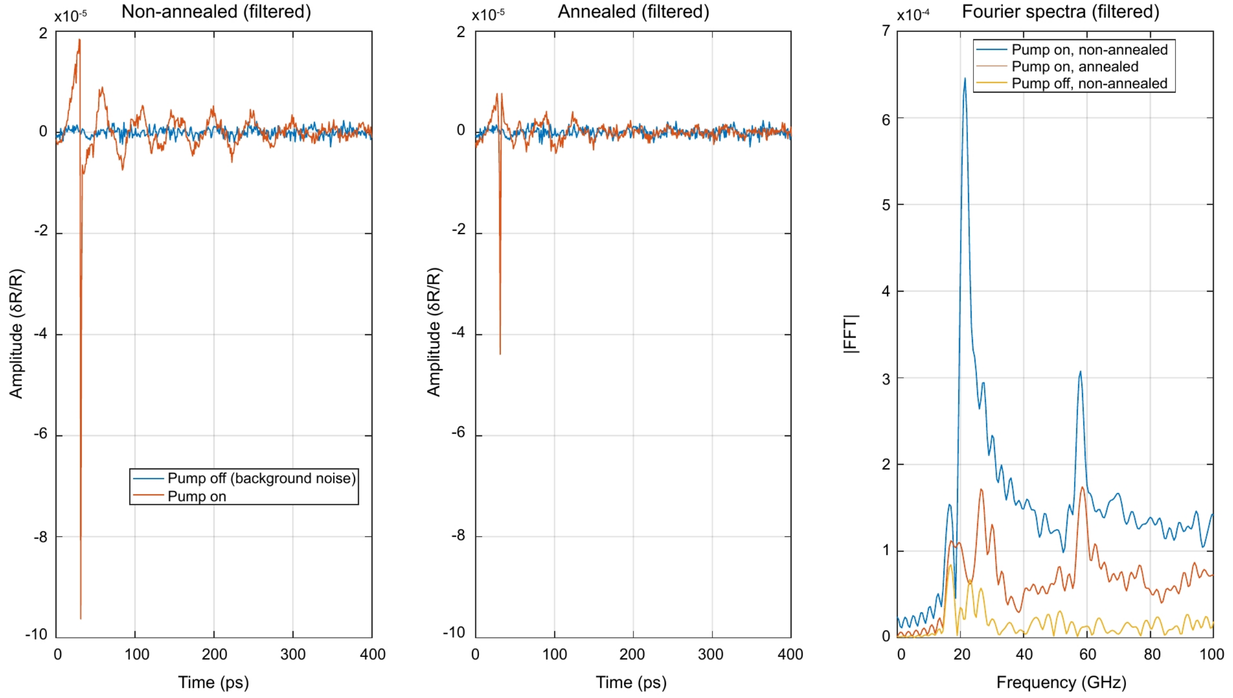

FIG. S15. High-pass filtered signal. **Left:** Non-annealed sample. **Middle:** Annealed sample. **Right:** Fourier transformed spectra.

where f_1 and f_2 are the frequencies of the first and second components in the signal ($\sim$20 GHz and $\sim$57 GHz respectively), both determined using the spectrum of Fig. S15, A_1 and A_2 are the amplitude of both components and ϕ_1 and ϕ_2 their phases. Finally, τ_{ac} is the time constant that characterizes the loss in acoustic amplitude due to consecutive reflections at the boundary, by transmitting this acoustic energy to the substrate. With good adhesion between the $SrTiO_3$ and SiO_2, a relatively large part of the acoustic energy is transmitted to the substrate, inducing a low value of τ_{ac}. For a bad adhesion, where most of the acoustic energy is kept inside the Au/Cr/$SrTiO_3$ assembly, the time constant is larger. Theoretically, two different time constants should be considered, one for each frequency (τ_1 and τ_2). However, the differences between τ_1 and τ_2 is quite weak ($\sim$10-20 ps) at the frequencies considered here and are below the error threshold on the estimation of the value of τ_{ac}. Furthermore, adding one more parameter for the fit decreases the reliability of the results. Therefore, the approximation $\tau_1 \approx \tau_2 = \tau_{ac}$ is considered here. The attenuation of the acoustic waves during the propagation inside the structure due to other effects (viscosity, scattering, etc.) is neglected here. The result is presented on Fig. S16 for both non-annealed and annealed case with their associated spectra. The black and blue curves

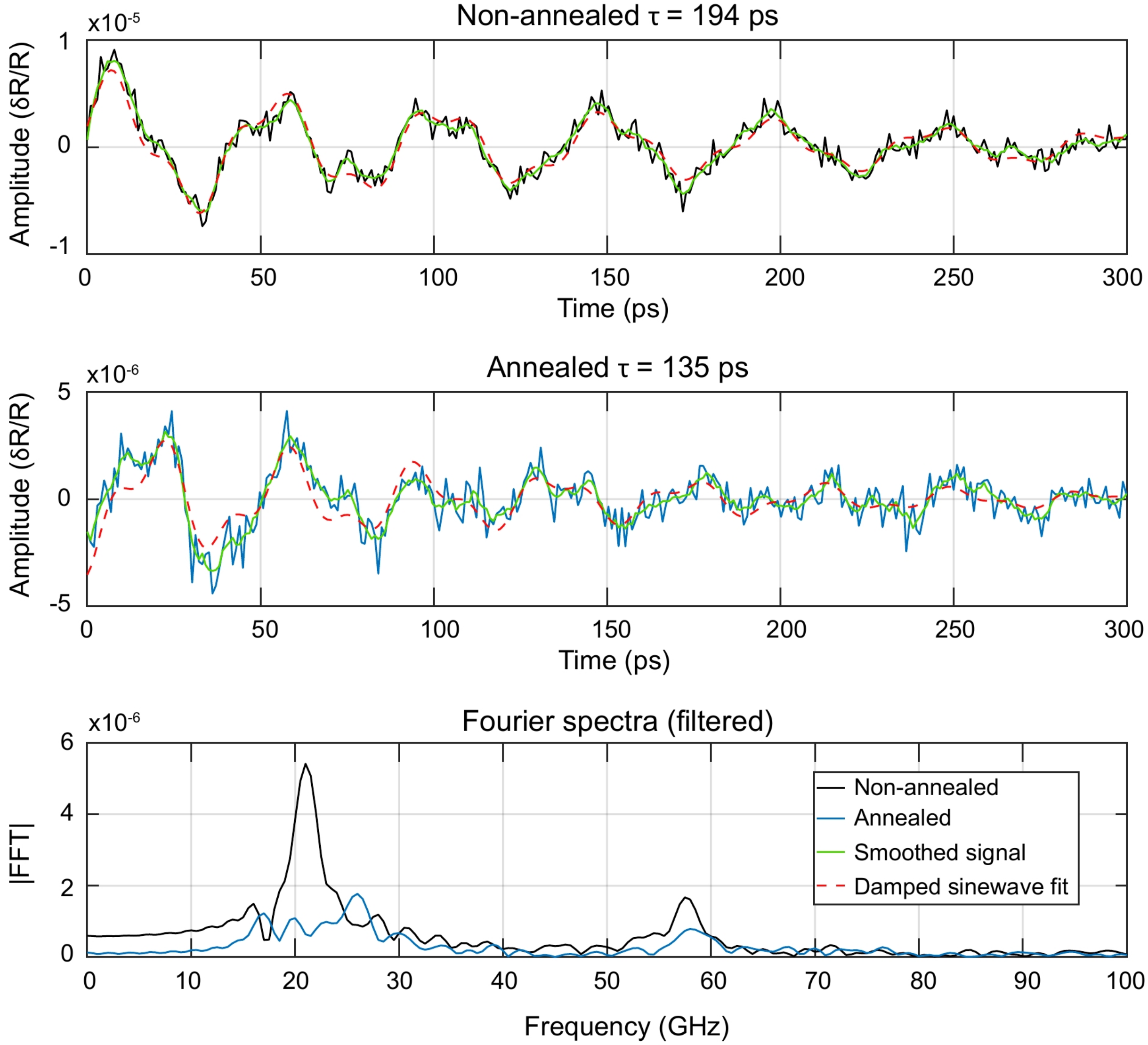

FIG. S16. Fit of the signals. **Top:** Non-annealed sample. **Middle:** Annealed sample. **Bottom:** Fourier transformed spectra.

correspond to the filtered raw data of non-annealed and annealed samples respectively, the green to the smoothed signal and the dashed red lines to the fit using Eq. S.1.

Calculations of the results

The theoretical resonance frequencies are calculated from the expressions of standing waves in a medium, as given by Greener *et al.*[8]:

$$f_{nU} = \frac{nc_{Leq}}{2h_{eq}}, \tag{S.2}$$

for an unbounded medium (total debonding case, i.e. free surface at each boundary) and

$$f_{nB} = \frac{(2n-1)c_{Leq}}{4h_{eq}}, \tag{S.3}$$

for a bounded medium (perfect adhesion case, i.e. loaded surface with continuity of stresses and displacement at the boundary with the substrate and free surface boundary condition at the other boundary of the structure). In the Eqs. S.2 and S.3, n is the order of the harmonic, c_Leq = $\frac{c_{L1}c_{L2}h_{eq}}{c_{L1}h_2+c_{L2}h_1}$ is the equivalent longitudinal wave velocity in the Au/Cr/$SrTiO_3$ assembly with c_{L1} and c_{L2} respectively the longitudinal velocity in the Au and $SrTiO_3$ layer. h_{eq} = h_1 + h_2 is the total thickness of the assembly with h_1 = 30 nm the thickness of the Au layer and h_2 = 80 nm the one of the $SrTiO_3$ layer. The chromium layer is here neglected due to its small thickness (3 nm) with respect to the total thickness of the structure and with respect to the acoustic wavelength (of the order of 100 nm).

The reflection coefficient is then deduce from the following formula, also used by Greener *et al.*[8]:

$$|A_p| = |A_0||R_{ac}|^p = |A_0|\left(e^{-\frac{2h_{eq}}{c_{Leq}\tau_{ac}}}\right)^p = |A_0|\left(e^{-\frac{1}{\tau_{ac}f_{1U}}}\right)^p, \tag{S.4}$$

where $|A_p|$ is the absolute value of the amplitude after p reflections of the acoustic wave inside the structure, $|A_0|$ the initial amplitude of the wave, $|R_{ac}|$ the absolute value of the reflection coefficient in amplitude. From the reflection coefficient, it is possible to deduce the longitudinal interfacial stiffness K_L, characterizing the adhesion between both materials at the interface[9]:

$$|R_{ac}| = \left|\frac{Z_2 - Z_s + \frac{j\omega Z_2 Z_s}{K_L}}{Z_2 + Z_s + \frac{j\omega Z_2 Z_s}{K_L}}\right|, \tag{S.5}$$

where Z_m = $c_{Lm}\rho_m$ the acoustic impedance of the medium m with ρ_m its density. Here, m = 2 for the $SrTiO_3$ and $m = s$ for the substrate (SiO_2). For all these calculations, the values of c_{L1}, c_{L2}, c_{Ls}, ρ_2, ρ_s have been taken from literature[10–12] and are given in Table S.XI

Figure S17 shows the variations of the reflection coefficient in amplitude by varying the interfacial stiffness at the frequency f_{1U} = 21 GHz.

Parameter	Au	$SrTiO_3$	Equivalent ($Au/SrTiO_3$)	SiO_2
**Longitudinal velocity c_L (m/s)**	2200	7900	4630	5800
**Density ρ(kg/m^3)**	-	5110	-	2650

TABLE S3. Values of the longitudinal wave velocities and densities of the different materials.

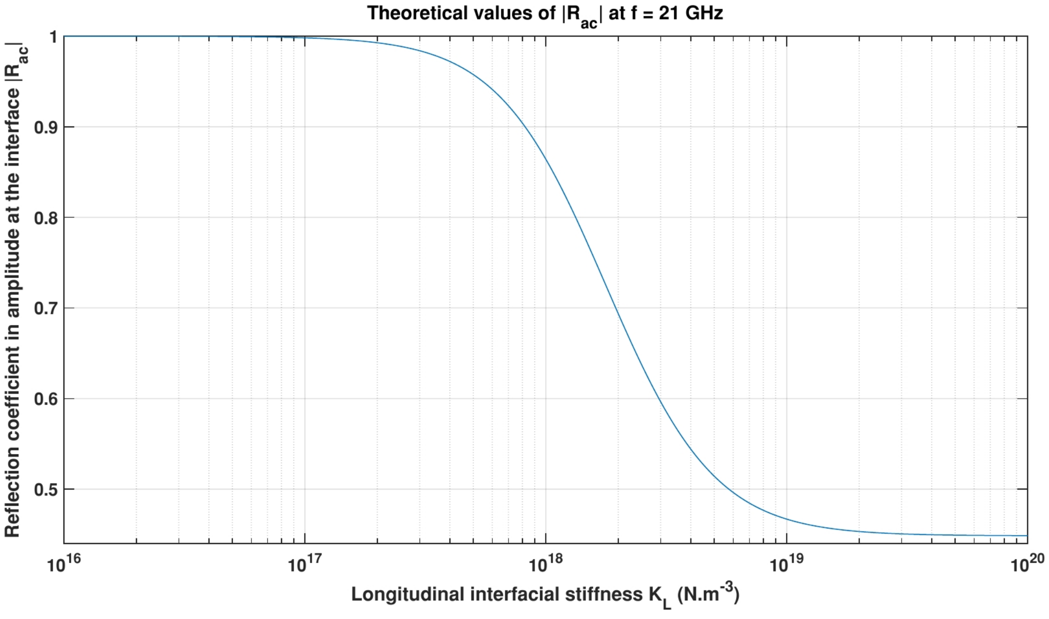

FIG. S17. Variations of the reflection coefficient with respect to the longitudinal interfacial stiffness.

The picosecond ultrasonics measurements were performed on 5 different annealed flakes and 4 different non-annealed flakes. Table S.XI gives the values of the frequencies and time constants for each measurements.

COMSOL simulations

The Finite Element Method simulations were performed using the heat transfer in solid and solid mechanics modules of the software COMSOL Multiphysics. The two modules were coupled together using the multiphysics thermal expansion module. The laser excitation was modeled as a heat source with similar characteristics than the pump laser pulses. The acoustic waves were detected using a point probe measuring the normal displacement at the surface of the 2D sample.

To simulate the total debonding, only an $Au/SrTiO_3$ sample was considered using free boundary condition at each end. To simulate a perfect adhesion case, an $Au/SrTiO_3/SiO_2$ sample was

	**Annealed flakes**			**Non-annealed flakes**		
	f_{1u} **(GHz)**	f_{3B} **(GHz)**	τ_{ac} **(ps)**	f_{1u} **(GHz)**	f_{3B} **(GHz)**	τ_{ac} **(ps)**
**1**	26	58	135.3	21	57.5	194.1
**2**	22.5	56	127.2	22.5	53	225.4
**3**	22.5	58	97.6	23	57	287.4
**4**	25	57	109.9	21	53.5	172.7
**5**	27.5	60	98.2	-	-	-
**Avg**	24.7	57.8	113.6	21.9	55.3	219.9
**Std**	2.2	1.5	17.1	1	2.3	50

TABLE S4. Values of the frequencies and time constants for each measurements on different flakes.

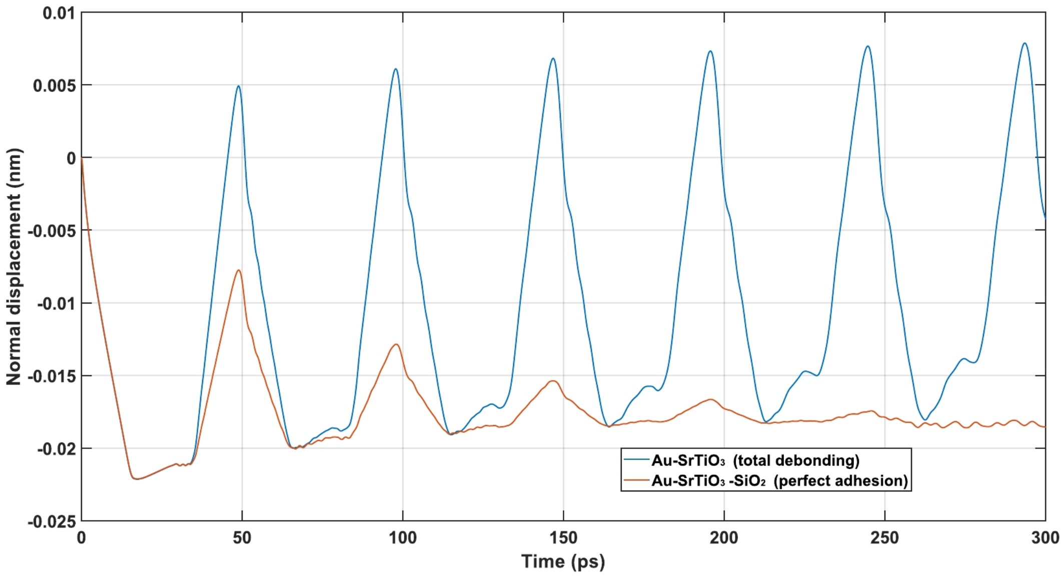

FIG. S18. Comsol simulation: comparison between the total debonding (blue) and perfect adhesion cases (orange).

considered with a "perfect" interface (corresponding to a case where $K_L \to \infty$ in Eq. S.5).

A triangular meshing was used with a maximum element size of 2.5 nm in Au and 4 nm in $SrTiO_3$ to have minimum ~15 elements per wavelength at 60 GHz in each materials. A coarser meshing (10 nm) was used in the SiO_2 substrate since the propagation of the waves in this material is not really of interest here. Its thickness has been chosen large enough to not have any backward

reflection coming from the bottom of the substrate. A time dependent study is then processed with a time step of 0.2 ps to get more than 60 samples per period of the acoustic waves at 60 GHz.

The results of the simulations are shown in Fig. S18. For the case with the substrate (corresponding to a perfect adhesion, orange curve on the Fig. S18), the reflection coefficient in amplitude $|R_{ac}|$ of 0.45 is found: this case corresponds to the limit $K_L \to \infty$, as shown in the Fig. S17. The case without the substrate (corresponding to a total debonding, blue curve in the Fig. S18), the reflection coefficient is equal to 1: this case corresponds to the limit $K_L \to 0$, as shown in Fig. S17.

REFERENCES

[1] A. Castellanos-Gomez, M. Buscema, R. Molenaar, V. Singh, L. Janssen, H. S. J. Van Der Zant, and G. A. Steele, "Deterministic transfer of two-dimensional materials by all-dry viscoelastic stamping," *2D Materials*, vol. 1, no. 1, p. 011002, 2014.

[2] F. Pizzocchero, L. Gammelgaard, B. S. Jessen, J. M. Caridad, L. Wang, J. Hone, P. Bøggild, and T. J. Booth, "The hot pick-up technique for batch assembly of van der waals heterostructures," *Nature communications*, vol. 7, no. 1, pp. 1–10, 2016.

[3] K. Kim, M. Yankowitz, B. Fallahazad, S. Kang, H. C. P. Movva, S. Huang, S. Larentis, C. M. Corbet, T. Taniguchi, K. Watanabe, *et al.*, "van der Waals heterostructures with high accuracy rotational alignment," *Nano letters*, vol. 16, no. 3, pp. 1989–1995, 2016.

[4] C. J. Shearer, A. D. Slattery, A. J. Stapleton, J. G. Shapter, and C. T. Gibson, "Accurate thickness measurement of graphene," *Nanotechnology*, vol. 27, no. 12, p. 125704, 2016.

[5] P. J. Zomer, M. H. D. Guimarães, J. C. Brant, N. Tombros, and B. J. Van Wees, "Fast pick up technique for high quality heterostructures of bilayer graphene and hexagonal boron nitride," *Applied Physics Letters*, vol. 105, no. 1, p. 013101, 2014.

[6] C. Thomsen, J. Strait, Z. Vardeny, H. J. Maris, J. Tauc, and J. J. Hauser, "Coherent phonon generation and detection by picosecond light pulses," *Physical review letters*, vol. 53, no. 10, p. 989, 1984.

[7] L. Liu, Y. Guillet, and B. Audoin, "Common-path conoscopic interferometry for enhanced picosecond ultrasound detection," *Journal of Applied Physics*, vol. 123, no. 17, p. 173103, 2018.

[8] J. D. G. Greener, E. de Lima Savi, A. V. Akimov, S. Raetz, Z. Kudrynskyi, Z. D. Kovalyuk, N. Chigarev, A. Kent, A. Patané, and V. Gusev, "High-frequency elastic coupling at the interface of van der Waals nanolayers imaged by picosecond ultrasonics," *ACS nano*, vol. 13, no. 10, pp. 11530–11537, 2019.

[9] M. Grossmann, M. Schubert, C. He, D. Brick, E. Scheer, M. Hettich, V. Gusev, and T. Dekorsy, "Characterization of thin-film adhesion and phonon lifetimes in Al/Si membranes by picosecond ultrasonics," *New Journal of Physics*, vol. 19, no. 5, p. 053019, 2017.

[10] K. Uozumi, T. Nakada, and A. Kinbara, "Sound velocity and internal friction in vacuum-deposited gold films," *Thin Solid Films*, vol. 12, no. 1, pp. 67–70, 1972.

[11] H. Muta, K. Kurosaki, and S. Yamanaka, "Thermoelectric properties of reduced and La-doped single-crystalline $SrTiO_3$," *Journal of alloys and compounds*, vol. 392, no. 1-2, pp. 306–309,

2005.

[12] D. Brick, E. Emre, M. Grossmann, T. Dekorsy, and M. Hettich, "Picosecond photoacoustic metrology of SiO_2 and $LiNbO_3$ layer systems used for high frequency surface-acoustic-wave filters," *Applied Sciences*, vol. 7, no. 8, p. 822, 2017.
